# Energetics of Glucose Metabolism: A Phenomenological Approach to Metabolic Network Modeling

**DOI:** 10.3390/ijms11082921

**Published:** 2010-08-12

**Authors:** Frank Diederichs

**Affiliations:** Marschweg 10, D-29690 Schwarmstedt, Germany; E-Mail: fwkh.diederichs@googlemail.com; Tel.: +49-5071-8521

**Keywords:** nonequilibrium thermodynamics, oxidative phosphorylation, efficiency, metabolic stability, glucose recognition

## Abstract

A new formalism to describe metabolic fluxes as well as membrane transport processes was developed. The new flux equations are comparable to other phenomenological laws. Michaelis-Menten like expressions, as well as flux equations of nonequilibrium thermodynamics, can be regarded as special cases of these new equations. For metabolic network modeling, variable conductances and driving forces are required to enable pathway control and to allow a rapid response to perturbations. When applied to oxidative phosphorylation, results of simulations show that whole oxidative phosphorylation cannot be described as a two-flux-system according to nonequilibrium thermodynamics, although all coupled reactions *per se* fulfill the equations of this theory. Simulations show that activation of ATP-coupled load reactions plus glucose oxidation is brought about by an increase of only two different conductances: a [Ca^2+^] dependent increase of cytosolic load conductances, and an increase of phosphofructokinase conductance by [AMP], which in turn becomes increased through [ADP] generation by those load reactions. In ventricular myocytes, this feedback mechanism is sufficient to increase cellular power output and O_2_ consumption several fold, without any appreciable impairment of energetic parameters. Glucose oxidation proceeds near maximal power output, since transformed input and output conductances are nearly equal, yielding an efficiency of about 0.5. This conductance matching is fulfilled also by glucose oxidation of β-cells. But, as a price for the metabolic mechanism of glucose recognition, β-cells have only a limited capability to increase their power output.

## 1. Introduction

In recent years mathematical modeling of metabolic pathways became increasingly important as a helpful tool for a deeper understanding of biochemical reaction networks in cellular metabolism and energetics. Besides older kinetic approaches, several new models were developed, whose flux equations contain driving forces, primarily to eliminate possible inconsistencies with the Second Law of Thermodynamics [1–9]. In two recently published articles [6,10], a new flux equation was introduced, which allows both, the description of kinetic flux behavior similar to Michaelis-Menten type equations [11], as well as the treatment of energetic relations known from methods of nonequilibrium thermodynamics (NET)[12–15]. Sometime later, a commensurable formalism (thermodynamic-kinetic modeling) was published by Ederer and Gilles [8,9].

In this study, the phenomenological character of equations is underlined, especially in relation to the realities of living cells. Equations are based on the concept of entropy production [16] and associated dissipation of free energy (dissipation function)[13]. Especially the dissipation function is particularly suited for the treatment of energetic relations of metabolic pathways like glucose oxidation. In this context, it seemed essential to test the applicability of flux equations to widely accepted results from NET. In this regard, the coupling behavior, not only of one single reaction but of the whole reaction network of oxidative phosphorylation (OP, [13,17–20]), is of outstanding interest.

One of the most remarkable results of heart physiology is that despite markedly increased power output and O_2_ consumption, energetic parameters of working hearts like the cytosolic ATP concentration ([ATP]_c_) are not appreciably lowered under these conditions [21–26]. This metabolic stability of the heart against increasing workloads is only partly understood. As a prerequisite for a strong power output, highly ordered structural elements seem to be indispensable. Saks and colleagues could show that the treatment of the intracellular environment as a diluted aqueous solution of electrolytes and metabolites stands in contradiction to experimental results [27]. According to these authors also the complex structural organization of the intracellular compartment into microcompartments like cytoskeletons and multienzyme complexes has to be considered. Moreover, for cells with a high power output such as cardiac muscle cells, it is required that free energy flow is controlled by a feedback from ATP utilization to ATP production [28].

In simulations presented here microcompartmentation is not addressed, only the cyto(sarco)solic compartment and the mitochondrial matrix are considered in simulations. However, even assuming these simplifying models, it is possible to demonstrate how ventricular myocytes (VMs) can cope with the problem of metabolic stability under conditions of high power output.

In comparison, β-cells are much smaller and less organized than VMs. Their power output is comparably low, and an increase of glucose concentration ([Glu]) is mandatory to activate insulin secretion. This increase of substrate concentration is not only needed as a stimulus [29,30], but is also necessary to fulfill the requirements of an increased ATP consumption during insulin release [10].

Both markedly different cell types use the same reactions of glucose metabolism, but with an appreciably different ability to gain free energy from these metabolic pathways. The solution of this problem may elucidate the control mechanism by which the flow of free energy of glucose oxidation becomes available for increased power output

## 2. Results and Discussion

### 2.1. Pathway Fluxes and Phenomenology

#### 2.1.1. Flux Equations

In previous articles (Diederichs, [6,10]) a new phenomenological type of flux equation to describe not only membrane transport reactions but also enzyme-catalyzed reactions as they occur in the living cell, was introduced. The new formalism combines some characteristics known from enzyme-catalyzed reactions with energetic relations of NET. They are comparable to several other linear relationships between corresponding flows and forces such as electric current flow, heat flow, or diffusional flow. The main difference to these latter phenomenological laws and to NET is brought about by introducing a variable instead of a constant conductance into flux equations. Conductances may be varied by substrate and ion concentrations as is known from enzyme kinetics [11], leading to the following expression:

(1a) $$J=L(c_{1},c_{2},\ldots )\times A\;(\mu \text{M}/\text{ms})$$

*A* (J/mol) denotes the affinity or driving force of a given reaction. It is defined as *A* = −*dG*/*dξ* (*G* = Gibbs free energy (J/mol), *ξ* = extent of reaction (mol)). *A* is not constant, but depends on the mass action quotient of variable nonequilibrium activities (≈concentrations). *L*(*c*_1_,*c*_2_,...) (μM/ms × mol/J) denotes the conductance of that reaction. It is treated here as a variable, which can be varied by concentrations of metabolites, cofactors, and inorganic ions like Ca^2+^ (c’s).

When applied to membrane transport reactions, flux *J* is given by

(1b) $$J=\alpha \times G(c_{1},c_{2},\ldots )\times \Delta \tilde{\mu }\;(\text{charged compounds})$$

or

(1c) $$J=\alpha \times G(c_{1},c_{2},\ldots )\times \Delta \mu \;(\text{uncharged compounds})\;\text{both in }(\mu \text{M}/\text{ms})$$

respectively. Both *G*’s denote conductances (in pS), *Δμ̃* and *Δμ* (both in mV) are the electro-chemical and chemical potential differences, respectively, of the transported substance. *α* represents a conversion factor converting electric current *I* into flux *J* (see Section 3).

Equation (1a) and (1b) are based on the following relationship derived from the Second Law of Thermodynamics: entropy production per unit time and volume of an irreversible chemical or biochemical reaction of a system (‘i’) like the cytosolic compartment is related to the reaction’s affinity and velocity by

(2a) $$\frac{d_{i}S}{dtV}=\frac{d\xi }{dtV}\times \frac{A}{T}\left(\frac{\text{J}}{\text{sLK}}\right)$$[16]

(*T* = absolute temperature (K), *V* = system volume (L), *d*_i_*S*/*dt* = entropy produced during time interval *dt*). For *T* = const., an equivalent form of this equation is given by

(2b) $$\frac{d_{i}S}{dtV}T=\frac{d\xi }{dtV}\times A,\text{or }\Phi =J\times A\left(\frac{\text{J}}{\text{sL}}\right)$$

(2c) $$J=\frac{d\xi }{dtV}\left(\frac{\text{mole}}{\text{sL}}\right)(\text{given here in }\mu \text{M}/\text{ms})$$

*Φ* is the dissipation function per unit volume, it is given in units of power dissipated per unit volume. It is identical to the heat dissipated by the irreversible reaction per unit time and volume. Analogous to electrical power *P* = *I* × *U* and current *I* = *L* × *U*, the expression of flux *J* then is given by Equation (1a). This latter formulation, which is used throughout this paper, may be advantageous in view of its similarity to relations known from techniques such as electric circuits and heat flow. It should be noticed, however, that *A* is not identical to a conjugated force *X*, which according to Equation (2a) is defined by *X* = *A*/*T*.

In the following, the relationship to a Michaelis-Menten type reaction is shown. The flux through an enzyme-catalyzed reaction may be expressed as

(3a) $$J_{M-M}=\frac{{\left[S\right]}^{k}}{{\left(K_{M}\right)}^{k}+{\left[S\right]}^{k}}\times V_{Enz}^{\text{max}}(\mu \text{M}/\text{ms})$$

(*V_Enz_*^max^ = maximal reaction rate of an enzyme-catalyzed reaction; *K_M_* = Michaelis-Menten (analogue) constant). For *k* = 1 Equation (3a) represents the well known Michaelis-Menten formula, and for *k* > 1, (3a) changes to Hill’s Equation [31]). Comparison of Equations (1a) and (3a) shows that the new formalism is obtained by replacing *V_Enz_*^max^ of (3a) by *L_Enz_*^max^ × *A*, yielding

(3b) $$J=\frac{{\left[S\right]}^{k}}{{\left(K_{M}\right)}^{k}+{\left[S\right]}^{k}}L_{Enz}^{\text{max}}\times A\;(\mu \text{M}/\text{ms})$$

with

(3c) $$L(c)=\frac{{\left[S\right]}^{k}}{{\left(K_{M}\right)}^{k}+{\left[S\right]}^{k}}L_{Enz}^{\text{max}}\;(\text{c}=[\text{S}],L_{Enz}^{\text{max}}=\text{const})$$

The *S* (*S* = substrate or effector) containing term of Equation (3a) and (3b) represents a dimensionless activation/inhibiting factor, which can be regarded as a concentration-dependent probability, by which the maximal conductance *L_Enz_*^max^ of an enzyme-catalyzed reaction may be reached. If several activating and/or inhibiting factors were involved with the catalytic process, then, according to probability calculus, these factors must appear as products in the respective flux Equation [6].

On the other hand, Equation (3a) can be regarded as a special case of flux equation derived from the more fundamental rate Equations (1a) or (3b). In Equation (3a) the variable term *L_Enz_*^max^ × *A* of Equation (3b) is replaced by the constant *V_Enz_*^max^ of (3a), which can be expressed as a product consisting of two constant factors *L_Enz_*^max^ and *A_const_*, respectively. In contrast to the variable affinity *A*, *A_const_* does not depend on variables given by the mass action quotient. Subsequently, all flux equations of the Michaelis-Menten type and of NET can be regarded as special cases of the new flux equation given in Equation (1a). The former contain variable conductances, but constant affinities, whereas the opposite is true for equations of NET; these contain constant conductances, but variable affinities.

The great advantage of flux equations containing both variable conductances as well as variable affinities, is that they are remarkably well-suited for metabolic network modeling. So flux control by activating or inhibiting effectors is made possible as it is known from enzyme kinetics [11]. The inclusion of driving forces has the advantage that energetic parameters like power output or efficiency of coupled processes as they occur in metabolic pathways can be calculated.

As was shown in reference [6], the new formalism is applicable to reactions under “far from” as well as under “near” equilibrium conditions. Far from equilibrium, the flux equation is dominated by activation factors. This is brought about by the logarithmic nature of *A*, which remains fairly constant in large logarithmic arguments. Under these conditions, the flux equation approaches Michaelis-Menten kinetics [6] and, therefore may be suitable to describe far from equilibrium reactions, which is demonstrated by present simulations (most reactions are far from equilibrium). Theoretically, it is possible to reverse such an irreversible flux, but only at extreme substrate and/or product concentrations which do not occur in living cells.

Under near equilibrium conditions, the flux equation becomes dominated by *A*, because the logarithmic function then approaches zero. In this region the slope of this function becomes markedly increased. Under these conditions, the flux equation approaches the NET formalism. This approach is all the more complete, the more the *K_M_* value of this reaction approaches zero.

**Figure 1:**
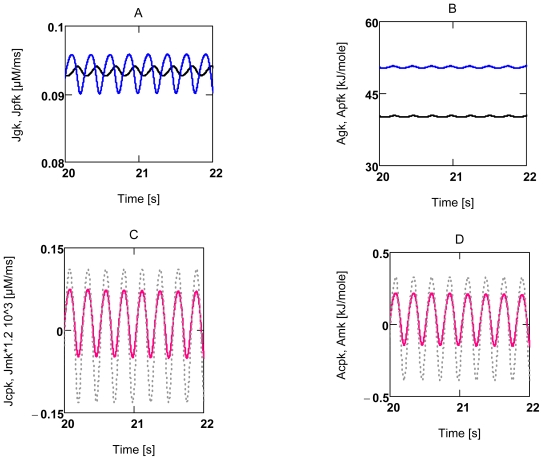
Oscillating fluxes at high and low affinities. **A:** (black) *J_GK_*, (blue) *J_PFK_*; **B:** (black) *A_GK_*, (blue) *A_PFK_*; **C:** (red) *J_CPK_*, (grey dots) *J_MK_*; **D:** (red) *A_CPK_*, (grey dots) *A_MK_*. Near equilibrium fluxes (panel C) are appreciably more influenced by respective affinities than fluxes far from equilibrium (panel A).

In Figure 1 the behavior of flux equations is demonstrated for conditions of [Ca^2+^]_c_ induced forced oscillations (see Section 2.3.2). At high *A*’s (panels A and B), flux oscillations are brought about mainly by activation factors, whereas at near zero driving forces, flux oscillations follow the oscillations of respective *A*’s about zero. The Second Law of Thermodynamics demands that the reaction rate of a spontaneously proceeding reaction goes against zero continuously. The oscillations about zero (Figure 1) do not violate this law, because these oscillations of the driving force are forced by the oscillating sarcosolic/cytosolic ADP concentration ([ADP]_c_).

#### 2.1.2. Constant *versus* Variable Conductances

To demonstrate the different behavior between pathway fluxes composed of constant and variable *L*’s respectively, a metabolic pathway composed of *n* in series enzyme-catalyzed reactions may be regarded. If, e.g., *L*_i_ is increased by a factor *f* at constant *L*’s, the pathway flux *J* expectedly would be increased by a factor *f′* < *f*. Then, according to Ohm’s law, the fractional increase of *J* is equal to the fractional increases of all *n* − 1 affinities at the *n* − 1 remaining conductances. Only the fractional change of *A_i_* at *L_i_* (fractional increase of *L_i_* is imposed and given by *f* − 1) must be different from all others. It is given by *ΔA*/*A*_0_ = (*ΔJ*/*J*_0_ − (*f* − 1))/*f*.

**Figure 2:**
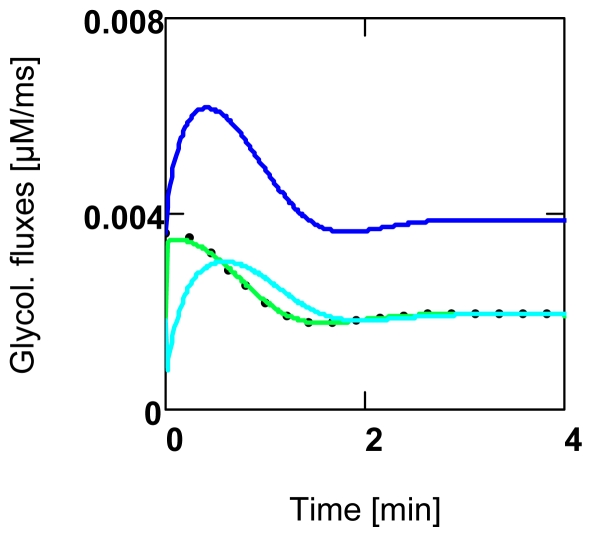
Behaviour of in series fluxes of glycolysis after a twofold increase (*f* = 2.0) of *L_GK_*^max^ · (black dots) *J_GK_*; (green) *J_Ald_*; (cyan) *J_Ti_*; (blue) *J_Ga_*. At steady state *J_Ga_* = 2 *J_GK_*; SIM*_GLY_*.

The above conditions of constant conductances are equivalent to a simple electric network, but are probably not compatible with a metabolic pathway. Since, under these latter conditions, variable enzyme activities determine conductances and thus might be responsible for a quite different behavior. The *n* − 1 fractional changes of affinities are not equal as is to be expected for constant conductances. This fact is associated with a remarkably different quality. Pathway fluxes containing variable conductances, after a given perturbation, can reach a new steady state much more rapidly than those having constant conductances. This is verified with a simple simulation (SIM_GLY_, see App. A14, the maximal conductance of the first reaction step, *L_GK_*^max,^ is increased by a factor *f* = 2.0). Under variable conditions a new steady state is reached already after 3.0 min (see Figure 2), whereas with constant *L*’s the new steady state develops only after 160 min (not shown).

In this context, it seems worth mentioning that Michaelis-Menten type formulations of pathway flux (const. *A*’s to yield *L_Enz_*^max^ × *A_const_* = *V_Enz_*^max^ =*const*. ), under the same conditions are unable to produce a new steady state. Obviously, in addition to variable conductances, the inclusion of variable driving forces in pathway fluxes is indispensible for the toleration of non-infinitesimal changes of conductances as they occur in living cells, for instance during activation of glucose metabolism, to increase power output.

With the new formalism, however, it is possible to describe metabolic networks as was shown recently with the known phenomena of stimulus-secretion coupling of β-cells [6,10]. In simulations the increase of only one single parameter, the glucose concentration, is necessary and sufficient to induce β-cell activation, as is known from experiments.

#### 2.1.3. Phenomenological Relations

In a metabolic pathway, which may be composed of a source and a sink and several in series enzyme-catalyzed reactions at steady state, the pathway flux can be expressed as

(4) $$J=\frac{1}{\frac{1}{L_{1}}+\frac{1}{L_{2}}+\frac{1}{L_{3}}+\ldots +\frac{1}{L_{n}}}(A_{1}+A_{2}+A_{3}+\ldots +A_{n})=L_{ov}A_{ov}$$

This follows from *Φ =* ∑*J_i_A_i_*, and at steady state *Φ = J*∑*A_i_* = *JA_ov_*. Furthermore, due to the similarity of the flux equation to Ohm’s law, in series conductances generate an equivalent conductance *L_ov_*. A constant overall affinity of the pathway *A_ov_* *=* ∑*A_i_* is then determined by the constant concentrations of metabolites of the source and sink, respectively. So, it is possible to contract several in series reactions of a given reaction sequence to one single step. In cellular metabolism this principle is used for metabolic channeling [32,33], which is brought about by aggregating several different enzyme molecules of a sequence to one catalytic complex. Because respective intermediates do not appear in solution, this may cause an appreciable increase of the overall conductance of the catalytic complex.

Since chemical and electro-chemical potentials are potential functions, their line integral over a closed path must vanish, *i.e*., Kirchhoff’s loop rule is applicable also to metabolic networks [2]. Kirchhoff’s second rule, the junction rule, however is not always valid. For instance, the aldolase reaction splits the first part of GLY into two equal fluxes, which merge again to the second part of GLY. The flux of this latter pathway is not equal to that of the first part, as is to be expected for an electric network (conservation of charge), but is increased by a factor of two. If, however a given flux is split up into several parallel fluxes, the junction rule is obeyed [2]. In any case, the law of mass conservation has to be fulfilled.

This latter fact has to be taken into account, when *A_ov_* and *L_ov_* are to be determined. Equation (4) is applicable only if a constant flux of given magnitude through the entire pathway is realized. With regard to GLY this would mean that for instance the flux of the second part (*J_Ga_*, A3) has to be transformed to yield the same magnitude as the flux of the first part. Since a given *Φ* can be expressed by any other reliable product of *J* and *A*, and consequently also by a product containing a *J* of desired magnitude, e.g., *J_GK_* (which is a measure of glucose utilization), an altered affinity has to be determined in such a way that *Φ* remains unchanged. This transformed affinity is given by

(5a) $$\vec{A}=\frac{\Phi }{J_{GK}}$$

and the associated transformed conductance by

(5b) $$\vec{L}=\frac{\Phi }{{\vec{A}}^{2}},\text{or }\vec{L}=\frac{J_{GK}}{\left|\vec{A}\right|}$$

Such transformed values have to be used, when the steady state pathway flux of a metabolic network containing one or several fluxes of different magnitude is to be determined. To develop a simulation of a metabolic network like coupled glucose oxidation, it is not necessary to know all parameters exactly. If glucose utilization and/or oxygen consumption are known from experiments, conductances can be adjusted for a maximal power output. Affinities of single reactions often are given or may be estimated from Gibbs energies of formation [Alb]. With known stoichiometric coefficients the simulation must yield correct results also with respect to thermodynamic constraints, even if the steepness of flux equations is not precisely met. This means that the use of adjusted constants like K_M_ values and other modeling parameters may suffice to fulfill reliability requirements.

#### 2.1.4. Flux Control Coefficients

In Metabolic Control Analysis (MCA) several dimensionless coefficients are defined, to quantify metabolic control at steady state [34]. One of these coefficients is the flux control coefficient *C_Ex_^J^*, which is defined as

(6) $$C_{Enz}^{J}=\frac{\Delta J_{x}}{J_{0}}/\frac{\Delta E_{x}}{E_{x0}}$$

(*E_x_* = one particular enzyme concentration of a metabolic pathway of several reaction steps in series; *ΔJ_x_* = change of pathway flux produced by a perturbation *ΔE_x_*/*E_x_* = *f* − 1). According to MCA, the sum of flux control coefficients ( $\sum C_{Enz}^{J}$) approaches 1.0, for ( *f* − 1) → 0.

To demonstrate changes of a pathway flux after a small perturbation, a simulation of GLY is used, in which the reduced nicotinamide adenine dinucleotide concentration ([NAD_red_]) is fixed (SIM_GLY_, A14). As a source and sink, glucose ([Glu]) and pyruvate ([Pyr]) concentrations (4 mM and 43 μM, respectively) are both held constant. The new steady states are produced by multiplying successively respective maximal conductances by the same factor *f*. In all cases a new steady state can be obtained, in which all individual fluxes of the pathway have changed by the same degree, including that with the imposed change. In addition, affinities and intermediates have changed, but to a different extent in each case. From the newly adjusted pathway fluxes *J*, the initial flux *J_0_*, and the imposed *f*, all flux control coefficients can be calculated. For *f* = 1.01 this yields *C_GK_^J^* = 0.1258, *C_Ald_^J^* = 0.0021, *C_Ti_^J^* = 0.00037, *C_G_^J^* = 0.0068, and *C_W_^J^* = 0.8623, yielding $\sum C_{Enz}^{J}=0.9974$.

In Figure 2 the behavior of all fluxes after increasing *L_GK_*^max^ of *J_GK_* (A1) by a markedly more pronounced perturbation (*f* = 2.0) is shown. After a new steady state has been reached, all fluxes are equally increased. To achieve this, the initially increased one (*J_GK_* from 2 × 1.8084 × 10^−3^) must decrease, whereas the other ones (*J_Ald_*, *J_Ti_*, and *J_Ga_*) must all increase until all individual fluxes of the first part of GLY (*J_GK_*, *J_Ald_*, and *J_Ti_*) are equal and a new steady state pathway flux is produced. All new fluxes are in fact increased, but the relative increase is much less than *f* − 1 (0.0694 compared to 1.0).

For conditions of constant conductances, the same simulation as described above but with constant *L*’s is used. For these conditions, the sum of *C_Enz_^J^* can be obtained analytically. It is derived from Equation (4) by building *n* fractional changes of pathway flux (*n* = number of individual in series fluxes of a pathway), of which each individual one contains in each case a conductance, which has been changed by *f*. Summarizing and dividing by *f* − 1 yields $\sum C_{Enz}^{J}(f)$ (not shown).

**Figure 3:**
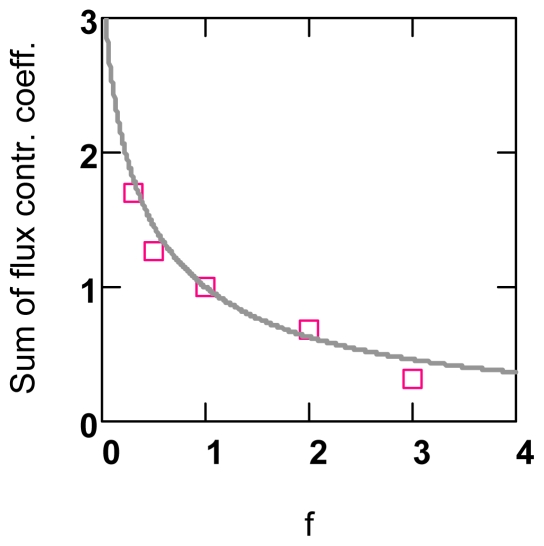
Sum of flux control coefficients $\sum C_{Enz}^{J}$ *versus* perturbation *f*. (red squares) results from simulation SIM*_GLY_* with variable conductances; (grey line) analytical curve $\sum C_{Enz}^{J}(f)$ for constant conductances.

For infinitesimal changes, $\sum C_{Enz}^{J}(f)\to 1.0$, and at *f* = 1.0, a singularity appears. For *f* > 1.0, this function is always smaller than 1.0, whereas the opposite occurs for *f* < 1.0 (Figure 3).

For variable *L*’s, the above relation cannot be solved analytically. The output values of the simulation, however, show that respective points follow roughly the analytical function. At small perturbations (*f* →1.0) the summation theorem seems to be sufficiently approached, as is shown above for *f* = 1.01. For larger changes, however, as they normally occur in cellular metabolism, deviations from 1.0 have to be expected (Figure 3).

In a more complex pathway like whole coupled glucose oxidation, coupled reactions and, in addition, load reactions (‘load’ usually designates the output affinity *A*_1_ of a cytosolic reaction coupled to ATP_c_ splitting) are involved, both of which may impair the constancy of *A_ov_*. As a result, fractional changes of the pathway flux are also influenced by a changed *A_ov_*. This is given by

(7) $$\frac{\Delta {\vec{J}}_{tot}}{{\vec{J}}_{tot0}}=\frac{\Delta {\vec{L}}_{tot}}{{\vec{L}}_{tot0}}\left(\frac{\Delta {\vec{A}}_{tot}}{{\vec{A}}_{tot0}}+1\right)+\frac{\Delta {\vec{A}}_{tot}}{{\vec{A}}_{tot0}}$$

According to Equations (5a) and (5b), transformed values have to be used, which are designated *A⃗_tot_* and *L⃗_tot_*, respectively, to describe more complex networks. Such influences become most pronounced, when uncoupled reactions are involved (see Section 2.3.1).

### 2.2. Energetic Coupling in Metabolism

In metabolism, the chemical free energy of one mole of hydrogen containing substrates like glucose is converted ultimately into transformed Gibbs energy of reaction of cytosolic ATP hydrolysis ( *Δ_r_G′*(*ATPc*) = −*A_ATPc_*) plus molar heat changes. This energy conversion is brought about by reaction-coupling and is widely described by NET [12–15]. The following known equations are usually used to describe coupling of a two-flux-system:

(8a) $$J_{1}=L_{11}X_{1}+L_{12}X_{2}$$

and

(8b) $$J_{2}=L_{12}X_{1}+L_{22}X_{2}$$

*J*_1_ and *J*_2_ designate the output and input fluxes (related here to *dξ*/*dtV*), respectively, *X*_1_ and *X*_2_ represent the output and input forces; here they are identical to driving forces or affinities *A*_1_ and *A*_2_, respectively. *L*_11_ and *L*_22_ are termed straight coefficients, and *L*_12_ is termed coupling coefficient. The degree of coupling is defined as

(8c) $$q=L_{12}/\sqrt{L_{11}\cdot L_{22}}$$

the phenomenological stoichiometry as

(8e) $$Z=\sqrt{L_{11}/L_{22}}$$

To show more transparently, how coupled and uncoupled fluxes work together, the above equations are given in an equivalent formulation for enzyme-catalyzed reactions and associated free energy conversions occurring in cellular metabolism [10]:

(8f) $$J_{1}=L_{c}((\lambda_{1}+1)A_{1}+A_{2})$$

and

(8g) $$J_{2}=L_{c}(A_{1}+(\lambda_{2}+1)A_{2})$$

or

(8h) $$J_{1}=L_{c}(A_{1}+A_{2})+L_{L1}\;A_{1}$$

and

(8i) $$J_{2}=L_{c}(A_{1}+A_{2})+L_{L2}\;A_{2}$$

*L_c_* represents the coupling conductance (= *L*_12_), whereas *λ*_1_ and *λ*_2_ are related to the leak conductances *L_L_*_1_ and *L_L_*_2_ by *λ*_1_ = *L_L_*_1_/*L_c_*, and by *λ*_2_ = *L_L_*_2_/*L_c_*.

The output affinity *A*_1_ is usually opposed to the input affinity *A*_2_, *i.e.*, *A*_1_ is negative, their sum however must be positive. At non-zero leak conductances, leak fluxes are produced in the direction of their respective driving forces. They are given by *J_L_*_1_ = *L_L_*_1_A_1_ (negative), and *J_L_*_2_ = *L_L_*_2_A_2_. Comparison of Equations (8a) and (8b) with Equations (8f) and (8g), yields *L*_11_ = *Lc* + *L_L_*_1_, and *L*_22_ = *Lc* + *L_L_*_2_. At *q* = 1.0 ( *λ*_1_ and *λ*_2_ are both zero, see Equation (8j)), *J*_1_ and *J*_2_ are identical and are given by *J_coup_* = *L_c_* (*A*_1_ + *A*_2_). The resultant driving force of in series affinities is given by *A_coup_* = *A*_1_ + *A*_2_. When *λ*_1_ and/or *λ*_2_ > 0 (*q* < 1.0), it can be taken from Equations (8f) and (8g) that now fluxes must be different, and that *J*_1_ becomes decreased by the negative leak flux *J_L_*_1_, whereas *J*_2_ is increased by *J_L_*_2_. Obviously, uncoupling is always brought about when such additional leak fluxes arise.

The degree of coupling in terms of *λ*’s can be derived from Equations (8f) and (8g):

(8j) $$\sqrt{\frac{L_{c}A_{1}}{L_{c}(\lambda_{1}+1)A_{1}}\frac{L_{c}A_{2}}{L_{c}(\lambda_{2}+1)A_{2}}}=\frac{1}{\sqrt{\left(\lambda_{1}+1)\;(\lambda_{2}+1\right)}}=q$$

The root has to be drawn, because from both flux ratios, *J_coup1_/(J_coup1_* *+ J_L_*_1_) and *J_coup2_/(J_coup2_* *+ J_L2_*), associated with affinities *A*_1_ and *A*_2_, respectively, only one resultant ratio can be associated with the flow through the coupling conductance *L*_c_. Thus, *q* represents the geometric mean of both ratios involved.

The efficiency of free energy conversion is given by

(8k) $$\eta =-\frac{\Phi_{1}}{\Phi_{2}}=-\frac{J_{1}A_{1}}{J_{2}A_{2}}$$[13]

or

(8l) $$\eta =-\frac{(\lambda_{1}+1)a+1}{1+\frac{\left(\lambda_{2}+1\right)}{a}}$$

*a* designates the force ratio *A*_1_/*A*_2_. An equivalent expression is

(8m) $$\eta =-\frac{x+q}{q+\frac{1}{x}}$$

with *x* = (*A*_1_/*A*_2_)*Z* (reduced force ratio). Maximal efficiency is given by

(8n) ηmax=q2(1+1-q2)2[13]

and efficiency at maximal power output by

(8o) $$\eta_{P\text{max}}=\frac{1}{2}\frac{q^{2}}{2-q^{2}}$$[20]

#### 2.2.1. Coupling in Oxidative Phosphorylation

During oxidative glucose metabolism hydrogen of glucose is transferred to oxidized hydrogen carriers like NAD_ox_, which in turn transfer their hydrogen to the respiratory chain of the mitochondrial inner membrane, where it reacts with oxygen to form H_2_O and reoxidized carrier. This reoxidation of redox carriers (NAD_red_ and FAD_red_, respectively) at the inner side of the mitochondrial inner membrane is coupled to the transport of protons from the mitochondrial matrix space to the outer side of the inner membrane. At the interface between the outer aspect of the inner membrane and the bulk solution of the intermembrane space (solution between inner and outer mitochondrial membranes) these protons may exchange with other cations like K^+^ ions of the intermembrane space, so that an electrochemical potential difference of protons over the inner membrane, *Δμ̃*_H_, is produced by coupled proton transport. It is composed of a chemical potential difference of protons over the inner membrane, *Δμ*_H_, plus an electrical potential difference, the membrane potential *Δ_m_φ*of the inner membrane, yielding in mV

(9) $$\Delta {\tilde{\mu }}_{H}(mV)=\frac{RT}{F}10^{3}\text{ln}\left(\frac{{\left[{\text{H}}^{+}\right]}_{m}}{{\left[{\text{H}}^{+}\right]}_{c}}\right)+z\Delta_{m}\varphi \;(\Delta_{m}\varphi \;\text{in mV},\text{z}=\text{charge number}=1.0)$$

For simulations presented in this and foregoing articles [6,10] it was assumed that the pH of the intermembrane space is identical to the cytosolic pH_c_ and that outwards pumped and inward flowing protons only affect matrix pH_m_, but have no effect on pH_c_ (e.g., *J_NA_* or *J_SY_*, A7 or A9). The same holds for proton symport and exchange reactions at the inner membrane: only pH_m_ can be changed, pH_c_ remains unaffected (e.g., *J*_Pi_ and *J*_HC_, see [2]). In contrast, *Δ_m_φ* is changed by both, in - and outgoing electrogenic membrane fluxes like *J_CU_* (see [2]) or *J_SY_*.

Such behavior might be realized for real mitochondria *in situ* by a further compartmentation of the intermembrane space. From recent morphological studies [35–38], it is known that cristae membranes form flat sacs with narrow openings to the intermembrane space. If proton cycling (outwards pumping and inwards flow) would preferentially occur at these structures, then primarily the luminal pH of cristae would be affected. This means that *Δμ̃*_H_ of the inner membrane might be transformed into a potential difference with a more pronounced (more negative) chemical component (*Δμ*_H_) and an appropriately less pronounced (less negative) electrical component (*Δ_m_φ*) at cristae membranes than at the rest of the inner membrane. A low buffering power of these regions would further increase this effect. Strauss *et al*. [36] could demonstrate that ATP synthase complexes are located preferentially at the rims of cristae sacs where they may be involved with shaping the rim structure. These morphological facts are consistent with the idea that pH_c_ under physiological conditions may be affected only to a negligible degree by proton fluxes across the inner membrane. So, an amount of *Δμ̃*_H_ × *n* (*J*) of energy is dissipated, when *n* moles of protons move without coupling across the inner membrane from the bulk phase of the intermembrane space to the bulk phase of the matrix.

Redox reactions (*J_NA_* and *J_FA_*, A7 and A8) are coupled to proton transport with a stoichiometry of 10.0 and 6.0 mol H^+^ per mol of NAD_red_ or FAD_red_ oxidized [18], respectively. Thus, 6.0 mol O_2_ and 12.0 mol reducing equivalents are consumed per mol of oxidized glucose. These chemical constraints are fulfilled by all simulations. But not all protons transported by *J_NA_* and *J_FA_* enter oxidative phosphorylation (OP). Some leak back into the matrix (*J_PL_*), some are needed for Ca/H exchange (*J_HCE_*), and some for the malate-aspartate shuttle (*J_MS_*). In addition, in real mitochondria H^+^ influx may be required for transhydrogenation. This membrane reaction, however, is not addressed in present simulations. The remaining proton flux is used by ATP synthase (*J_SY_*) and ATP/ADP exchange (*J_AE_*) plus H/Pi symport (*J_Pi_*). At steady state, the stoichiometry of coupled *J_SY_* is assumed to be 3.0 mol H^+^ per mol of matrix ATP (ATP_m_) produced [18]. ATP_m_ is transferred into the cytosol and ADP_c_ plus Pi are delivered to the matrix by two further in series reaction steps (via *J_AE_* and *J_Pi_*, respectively). Both reactions are coupled to inward transport of 1.0 mol of positive charge (via *J_AE_*) and 1.0 mol of electrically neutralized protons (by symport via *J_Pi_*) per mol of exchanged ATP_m_, *i.e.*, one additional *Δμ̃*_H_ is consumed to exchange ATP_m_ from the matrix with ADP_c_ and P_i_ from the cytosol. Thus, under near static head conditions, OP would require 4.0 *Δμ̃*_H_ to overcome in series affinities of ATP synthesis in the matrix (−*A_ATPm_*, A13) and of ATP exchange (*A_AdPi_*, A13). Under more physiological conditions, however, ATP_c_ consumption occurs permanently to drive load reactions of the cytosol. Mainly these reactions lower the ratio *A_ATPc_* *|****Δ****μ̃*_H_ below 4.0 (if ATP production by GLY and CAC were included, this value would be slightly higher).

When ATP splitting by load reactions is added, the whole process may be considered as two interconnected cycles: a proton cycle *J_H_^coup^* coupled to an ATP cycle *J_ATP_^coup^* through the ATP synthase reaction, ATP/ADP exchange, and H/Pi symport. At steady state (all individual fluxes of a given cycle are of same magnitude), both cyclic flows produce no dissipation (or entropy), because their respective sums of affinities taken over a closed path in both cases must be equal to zero.

In the above derivation proton fluxes from proton sources to proton sinks at the outer and inner sides of the mitochondrial inner membrane are not included in calculations. Under real conditions, however, in order to allow proton cycling, such fluxes must be present, and thus, also a certain amount of dissipation of free energy. In addition, during ATP cycling free energy is dissipated by diffusional fluxes of ATP, ADP, and P_i_ species (see Section 2.4). It can be concluded, therefore, that under real conditions OP must be always slightly uncoupled, even if all respective inner membrane reactions of OP would proceed at *q* = 1.0.

The flux ratio of coupled cycles, *J_H_^coup^* *| J_ATP_^coup^* must always be equal to 4.0 under totally coupled as well as under uncoupled conditions, as long as the ATP synthase reaction (*J_SY_*) remains coupled. In addition to the coupling between cycles, the proton cycle is coupled to *J_Rop_^o^*, and the ATP cycle to *J_Wop_^ld^* (A13), respectively. Both respective affinities, *A_R_^o^* (input force) and *A_W_^ld^* (negative output force), are the only forces, which do not vanish at steady state cycling.

The reaction sequence of OP is given by the following in series reactions: *J_R_^o^* denotes the resultant input flux of OP of parallel input reactions (fraction Q_H_ of *J_NA_* plus *J_FA_*, A13a). From total *J_R_^o^* only the coupled fraction without leak flux can be used for proton pumping, while *J_R_^h^* (coupled flux minus leak flux) is the effective proton efflux. At steady state it is equal to proton influx *J_SY_^h^* plus (*J_AE_* − *J_CAC_*). From total *J_SY_^h^* only the coupled fraction is used for ATP_m_ synthesis, but only *J_SY_^p^* (coupled flux minus leak flux) is exported from the mitochondrion to produce ATP_c_. It must be equal to (*J_AE_* − *J_CAC_*) and coupled *J_R_^o^*.

#### 2.2.2. Indirect Uncoupling

Equation (10) cannot give any information about the mechanisms of coupling and uncoupling, respectively. However, from their derivation [39] it follows that uncoupling leak fluxes must flow through the coupling device, *i.e.*, through the respective multi-enzyme complex involved, because they are added to input and output fluxes, respectively. In contrast to this direct uncoupling an indirect uncoupling may occur through additional parallel leak fluxes like the proton leak flux *J_PL_* at the inner mitochondrial membrane, or the flux of Na^+^ and K^+^ ions through sodium and potassium channels or other Na^+^ and/or K^+^-permeable channels of the cell membrane. The relevant coupled reaction for these latter uncoupling fluxes is given by the reaction sequence of the Na-K pump. Also Ca^2+^pumps must be mentioned in the context of such pump and leak fluxes. Remarkably, dissipation of free energy is used further by these systems for signal transduction.

In principle, to avoid static head production, coupled reactions are controlled in addition to parallel leak fluxes by their own substrates. For instance, Na-K pumps are kinetically controlled by [Na^+^]_c_, and Ca^2+^ pumps by [Ca^2+^]_c_, so that pump fluxes slow down when substrate concentrations become sufficiently lowered.

#### 2.2.3. Stucki’s Conductance Matching

Stucki suggested that energy coupling through OP may be described as a two-flux-system in the same way as for a single coupled reaction [40]. He identified the oxygen utilizing redox reaction as the input and the ATP_c_ yielding reaction as the output flux. Including an additional attached ATP_c_ consuming load reaction, he could show that the dissipation function (entropy production in his article) of such a system possesses a minimum at a reduced force ratio given by *x*_min_ = − *q*/(1 + *L*_33_/*L*_11_) (*x* = *A*_1_/*A*_2_ × *Z*), *L*_33_ and *L*_11_ are Stucki’s load and input conductances, respectively). Equating this value with the *x* coordinate of maximal efficiency, $x_{\text{max}}=-q/\left(1+\sqrt{1-q^{2}}\right)$, shows that a minimal dissipation and a maximal efficiency would coincide, if the ratio of the load (*L_33_*) and the input (*L_11_*) conductance of whole OP fulfilled the condition $L_{33}/L_{11}=\sqrt{1-q^{2}}$. This relation was termed conductance matching by Stucki [40]. It is derived for uncoupled systems only, because under totally coupled conditions the above relationship requires that *L*_33_ vanishes. This, however, would generate a static head state, which is not compatible with conditions of metabolizing cells.

It seems already worth mentioning that all simulations presented here and those published recently [6,10] do not adjust to such a value. Moreover, they all allow total coupling without producing a static head.

To prove Stucki’s hypothesis of conductance matching, a simulation of OP fuelled by pyruvate was developed (SIM_OP_*^Pyr^*, A16). Under such conditions OP may be better comparable to conditions of isolated mitochondria, especially because activation of PFK by cytosolic adenosine monophosphate concentration [AMP]_c_ is absent.

**Figure 4:**
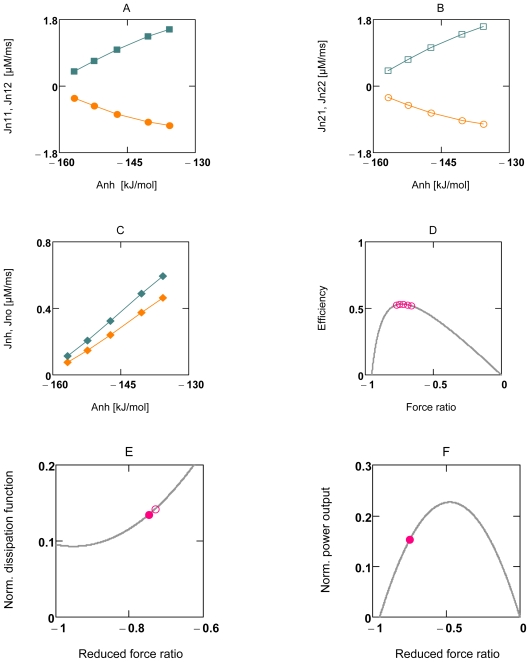
NET analysis of *J_NA_* as a two-flux-system. *J_NA_^h^* and *J_NA_^o^* are both uncoupled by *λ_n_^h^* = *λ_n_^o^* *=* 0.05 (*q_n_* = 0.952, *Z_n_* = 1.0). **A:** (filled circles) Jn11, (filled squares) Jn12, both *versus* Anh; **B:** (open circles) Jn21, (open squares) Jn22, both *versus* Anh; **C:** (orange diamonds) Jnh, (green diamonds) Jno, both *versus* Anh; **D:** efficiency *versus* force ratio *A_NA_^h^| A_NA_^o^* (open circles) results from simulation; (line) analytical results according to (10 *ℓ*); **E:** normalized dissipation function *versus* reduced force ratio *x*, (filled circle) result from simulation, (open circle) result from simulation, but at *x_max_* (abscissa of maximal efficiency), (line) analytical curve according to A20a; **F:** normalized power output *versus x*, (filled circle) result from simulation, (line) analytical curve according to A20b; SIM_OP_^Pyr^.

**Figure 5:**
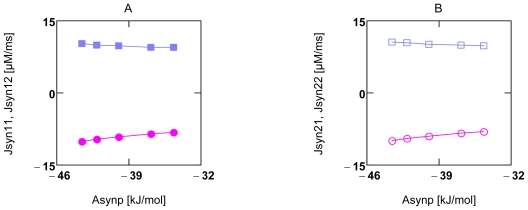

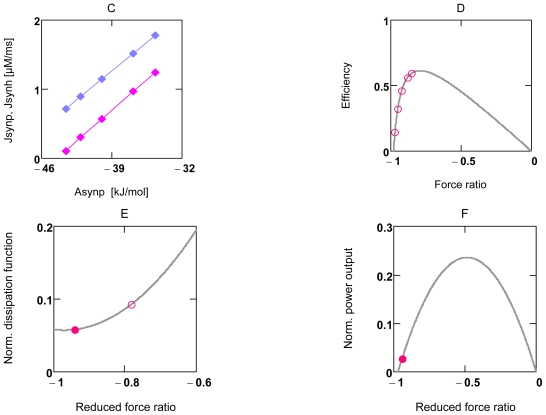
NET analysis of *J_SY_* as a two-flux-system. *J_SY_^p^* and *J_SY_^h^* are both uncoupled by *λ_s_^p^* *=* 0.02 *λ_s_^h^* = 0.04 (*q*_s_ = 0.971, *Z_s_* = 0.99). **A:** (filled circles) Jsyn11, (filled squares) Jsyn12, both *versus A_SY_^P^* (Asynp); **B:** (open circles) Jsyn21, (open squares) (Jsyn22), both *versus A_SY_^P^* (Asynp); **C:** (red diamonds) Jsynp, (green diamonds) Jsynh, both *versus A_SY_^P^* (Asynp); **D:** efficiency *versus* force ratio (*A_SY_^P^| A_SY_^h^*), (open circles) results from simulation; (line) analytical results according to (8l); **E:** normalized dissipation function *versus* reduced force ratio *x*, (filled circle) result from simulation, (open circle) result from simulation, but at *x*_max_, (line) analytical curve according to A20a; **F:** normalized power output *versus x*, (filled circle) result from simulation, (line) analytical curve according to A20b; SIM_OP_^Pyr^

At first, it is demonstrated that experimental results of Stucki can be principally reproduced by the present simulation of OP. Figures 4 and 5 show that under uncoupled conditions a linear relationship exists between *J*_1_ and *X*_1_ (*J_NA_^h^* and *A_NA_^h^*, and *J_SY_^p^* and *A_SY_^p^*, respectively), as well as between *J*_2_ and *X*_1_ (*J_NA_^o^* and *A_NA_^h^*, and *J_SY_^h^* and *A_SY_^p^*, respectively, A7 and A9). From this linear behavior Stucki concluded that for a given *q_ov_* (overall value of *q* for whole OP), *X_1_* may be the only variable of this two-flux-system, and that all other parameters, *i.e*., *X_2_*, *L_11_*, and *L_12_* are constant and can be derived from experimental results [40].

The simulation demonstrates, however, that linearity under such conditions can be produced with variable conductances and variable forces as well. It can be taken from Figures 4 and 5 (panels A and B) that the fluxes *J*_11_ and *J*_12_, whose sum yields *J*_1_, are slightly curved with opposite curvature, but that their sum yields a straight line. The same holds for *J*_2_. The slight deviation from linearity shows that some results of the simulation only approximately obey flux equations of NET. Under coupled conditions, fluxes *J_1_* and *J_2_* are equal and linear (not shown).

Figures 4d and 5d demonstrate that uncoupling of individual reactions of OP fulfill the analytical efficiency equation (10). Panels D-F of Figure 4 show that for this individual two-flux-system (*A_NA_* is far from equilibrium) efficiency is close to the maximum, but that dissipation is not minimal, whether at the reduced force ratio *x_max_* (where efficiency is maximal), nor for that obtained from the simulation. Power output of this reaction is 67.5% at *q_n_* = 0.952. In contrast, panels D-F of Figure 5 show that efficiency of this near equilibrium reaction (*A_SY_* is near zero) is not maximal. Dissipation is close to a minimum for the *x* value obtained from the simulation, but not for the *η*_max_ related *x*. As can be expected, power output for this near equilibrium reaction is much lower, only 11.7% at *q_s_* = 0.971.

The results of Figures 4 and 5 demonstrate that individual reactions of OP fulfill exactly the equations of NET. If, however, whole OP were formulated as a two-flux-system with *A_R_^o^* as input force, and −*A_ATPc_* as output force, *A_W_^p^* *=A_ATPc_* (positive) would be the input force of the attached load reaction. Because both affinities cancel, the reduced force ratio *x* of such a system would be zero, so that the dissipation function would be given merely by the term *L_OP_*(*λ*2*_ov_* + 1)(A*_R_^o^*)^2^ (A19b) For this expression, efficiency cannot be formulated as a function of *x*. It must be zero, because the output force is zero under all conditions. A conductance matching, as demanded by Stucki, therefore does not exist in simulations presented here, and, since the reaction sequence of ATP formation and ATP consumption must be present as an indispensible part of energy metabolism, this mechanism may also not be realized in a living cell.

Furthermore, from a teleological perspective, Stucki’s conductance matching seems highly unlikely to occur in energy metabolism of living cells, since such behavior would lead to the paradoxical situation that most favorable conditions of energy conversion – namely power output without losses of free energy by uncoupling – must result in cell death by the inevitable emergence of a static head.

When dissipation functions are formulated up to −*A_ATPc_* *or A_W_^ld^*, all forces but the input force (*A⃗_R_^o^*) and the output force (−*A_ATPc_* or *A_W_^ld^*, respectively) must vanish to describe the involved reactions as a two-flux-system. Although this is possible, unreliable results are produced, which often show negative *λ_ov_*’s and a *q_ov_* > 1.0, respectively. This might be caused by the cyclic mode of operation of the coupling system. It is concluded, therefore, that it is impossible to describe OP as a two-flux-system according to NET, although individual reactions of this complex coupling system may fulfill those equations.

### 2.3. Activation of Power Output in Glucose Metabolism

In the following, how the pathway of glucose oxidation may react on increasing demands of power output including uncoupling will be demonstrated. Two very different cell types—the ventricular myocyte with a high potential of power output, and the pancreatic β-cell with a relatively low power output—will be compared.

#### 2.3.1. Ventricular Myocytes

Hexokinase of heart muscle has a very low K_M_ value compared to that of glucokinase of β-cells. It is this kinetic difference of the first glycolytic reaction step, which is necessary to allow high pathway fluxes of glucose oxidation in muscle cells. In addition, the low K_M_ prevents that the intracellular glucose concentration ([Glu]) might become limiting as in β-cells (see below). Because [Glu] activation of hexokinase is saturated at resting concentrations ([Glu] = 4.0 mM), its conductance cannot vary which makes it more difficult if not impossible to develop a new steady state after a perturbation. This can be avoided by introducing a feedback inhibition of hexokinase by the concentration of glucose-6-phosphate ([G6P]), which in simulations can be expressed by the concentration of fructose-6-phosphate ([F6P]), since both intermediates are virtually at equilibrium through the phosphoglucose isomerase reaction (A2). Such a feedback can also avoid an extreme increase of [G6P] and [F6P]. So [F6P] appears as an additional variable in simulations of glucose oxidation in VMs. As a consequence, and in contrast to β-cell simulations, the PFK reaction is incorporated as an individual reaction step (not part of several in series reactions contracted to one single step as in β-cells) into the glycolytic reaction sequence of VMs. It is activated by [F6P] and in addition by [AMP]_c_, and is inhibited by [ATP]_c_. Also the pyruvate kinase (PK) reaction, which is part of several contracted in series reactions, is inhibited in VMs by [ATP]_c_ (A3).

To see which reactions of the glucose oxidation pathway must be activated to increase power output of ATP splitting reactions of the cytosol, MCA has been applied to these pathways. A simultaneous change of all conductances yields a value of *C^J^* = 1.0. When conductances are changed successively, *C^J^* does not reach 1.0. A pronounced change can already be obtained by changing simultaneously merely two conductances, that of *J_W_* (*G_Wser_*^max^, *L_Wcrbr_*^max^, *L_Wb_*^max^*,* A4 – A6), and that of *J_PFK_* (*L_PFK_*^max^; A2). For *f* = 2.0, *∑C_Enz_^J^* under these conditions reaches a value of 0.8725. Interestingly, this value can be appreciably increased further to 0.9757 by including the indirect uncoupling flux *J_PL_*. In contrast, when all remaining conductances of these pathways are increased simultaneously by *f* = 2, this yields a *∑C_Enz_^J^* value of 0.0112 only. It seems noteworthy that also a direct uncoupling can appreciably augment *∑C_Enz_^J^*. For instance, increasing conductances of only [Ca^2+^]_c_ dependent load reactions by *f* = 1.1, yields a *∑C_Enz_^J^* of 0.248. When under the same conditions the ATP synthase reaction is slightly uncoupled (*λ_sp_* = *λ_sh_* = 0.01), a *∑C_Enz_^J^* of 2.77 is obtained. This large increase of the sum of control coefficients by uncoupling is brought about by an appreciable increase of *A⃗_tot_* (see Equation (7)). Therefore, if a *∑C_Enz_^J^* >> 1.0 were found with a pathway containing coupled reactions, this may indicate a significant uncoupling.

The above results of control analysis show that increasing only two conductances may be sufficient to appreciably increase pathway flux and thus, also power output. A simultaneous increase of all maximal conductances is not necessary; the variability of conductances obviously is able to produce the demanded increase of whole pathway flux. Moreover, also fluxes through *J_AE_* and *J_Pi_* must not be specifically activated. Ca^2+^ activation of pyruvate dehydrogenase (PDH), and dehydrogenases of the citric acid cycle (fluxes *J_PDH_* and *J_CAC_*, respectively) is virtually without effect on power output and O_2_ consumption in present simulations, because respective conductances (*L_PDH_*^max^ and *L_CAC_*^max^) are adjusted to rather high values.

In real VMs, these conductances might be appreciably lower, so that a [Ca^2+^]_m_ activation becomes indispensable at an increasing power output [23,24,41–43]. Also ATP synthase of VMs is known to be activated by [Ca^2+^]_m_ [44]. In present simulations, activation of ATP synthase (*J_SY_*) by [Ca^2+^]_m_ cannot increase power output, but reduces dissipation of free energy of this reaction. Beside these latter effects of [Ca^2+^]_m_, mitochondrial Ca^2+^ fluxes may be needed to synchronize free energy flows of several mitochondria during contraction cycles. Otherwise an optimal interplay of all involved reactions might not be guaranteed.

In the present simulation as well as in real VMs, important load reactions like muscular contraction or Ca^2+^ transport by SERCA pumps are activated by an increase of [Ca^2+^]_c_. This in turn leads to an increase of ATP_c_ consumption with a concomitant elevation of [ADP]_c_ and [Pi]_c_. Because the adenylate kinase (AK) reaction is near equilibrium, this means that also [AMP]_c_ must increase and PFK becomes activated, with the result that all pathway fluxes associated with glucose oxidation become activated too. So activation of ATP_c_ utilization is followed by an appropriately adjusted ATP_c_ delivery. In this way load reactions not only deliver useful work, but concomitantly generate [AMP]_c_, which quasi as a third messenger activates ATP_c_ production, so that a drastic decrease of [ATP]_c_ and associated *A_ATPc_* may be prevented, even under conditions of a markedly increased power output. Present simulations demonstrate that the increase of only [Ca^2+^]_c_ dependent load conductances and [AMP]_c_ dependent *L_PFK_* is needed, to switch from low to a high power output.

O_2_ consumption of working hearts shows linear dependence on mechanical power output over a wide range of delivered power [23–25]. Such behavior can be simulated, supposing that pressure development of whole hearts is mechanically produced by the contractile machinery of single VMs, *i.e.*, by ATP- driven cross-bridge cycling. To formulate this process as a two-flux-system, a molar torque *τ*^m^ (generated by cross-bridge cycling) is defined as output force *A_τ_* coupled to the input force *A_ATPc_* (A5). An increasing pressure development of the activated heart is considered in simulations through a [Ca^2+^]_c_ dependent change of *τ*^m^. From NET it is clear that increasing *A_τ_* must reduce the resultant force *A*_coup_, so that the associated flux must be reduced too. This does not only decrease velocity of contraction, but also increases efficiency of this reaction.

**Figure 6:**
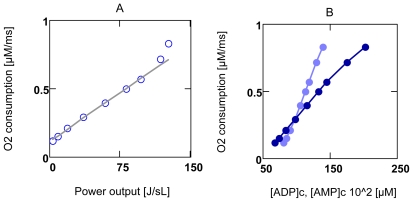
O2 consumption *versus* power output of contraction. **A**: (circles) results from simulation; (line) regression line: *J_O_*_2_ = 4.7446×10^−3^ × *P_W_^τ^+*0.116, r = 0.9993 (without the top two points); **B**: (light-blue) O_2_ consumption *versus* [ADP]_c_, (dark-blue) O_2_ consumption *versus* [AMP]_c_. SIM*_GlOx_*.

Figure 6A shows that results of simulation are consistent with experiments: over a wide range of power *P_Wτ_*,= −*Φ_Wτ_*= −*J_Wτ_A_Wτ_*, *J*_O2_ depends linearly on this variable. Only at very high [Ca^2+^]_c_’s, O_2_ consumption begins to be larger than would be expected from linearity. Panel B shows that *J*_O2_ depends linearly on [ADP]_c_ and [AMP]_c_. These results of simulations, however, do not support the conclusion that O_2_ consumption may be activated by [ADP]_c_ at the level of ATP/ADP exchange of mitochondria. Even a tenfold increase of this flux conductance (*L_AE_*^max^) could not further increase O_2_ consumption, which underlines above mentioned results of MCA. With permeabilized cardiomyocytes it could be shown [28] that the activation effect may be caused by a limited availability of ADP for OP, since ADP^3−^ cannot permeate with sufficient rapidity the voltage dependent anion channel (VDAC) [45]. The demonstrated Pi activation of O_2_ consumption [46,47] presumably cannot be explained by a limited availability of H_2_PO^4−^, since, in contrast to ADP^3−^, this Pi species may easily permeate the VDAC pore of the outer membrane. It was suggested that Pi may activate dehydrogenases of the matrix and in addition may be needed to activate electron transport of the respiratory chain [46].

But even if the above mentioned activation effects of [ADP]_c_ and/or [Pi]_c_ were operative also in coupled glucose oxidation of real VMs, an activation of whole pathway flux is absolutely necessary to deliver free energy under conditions of an increased consumption of free energy. Since it is not stored sufficiently in glucose metabolism itself including OP, it must be delivered by other stores, that is, mainly from the extracellular space in the case of VMs. So the controlled flow of free energy through the whole metabolic pathway is necessary to satisfy changing free energy demands. As is demonstrated with simulations, the most effective points of control are given by [Ca^2+^]_c_ dependent load reactions and PFK. The flow of information from load to delivery is brought about by the feedback via AK of [AMP]_c_ on PFK.

#### 2.3.2. Forced Oscillations

**Figure 7:**
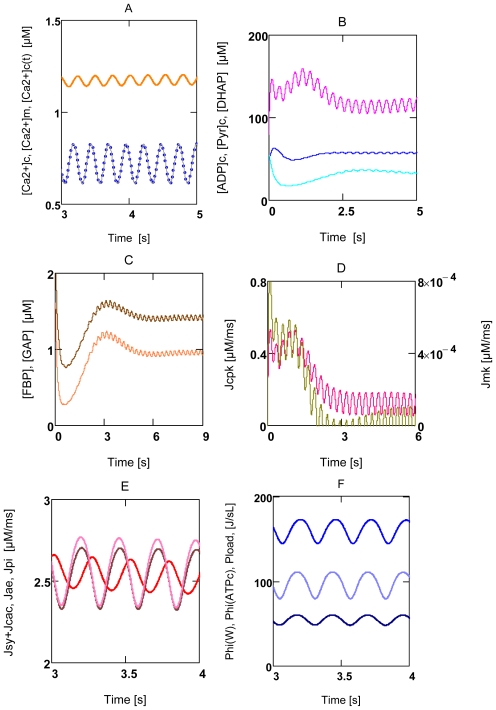
Forced oscillations produced by [Ca^2+^]_c_ oscillations according to A18. **A:** (grey)[Ca^2+^]_c_, (orange)[Ca^2+^]_m_, (blue points) oscillations according to A20; **B:** (red)[ADP]_c_, (cyan)[Pyr]_c_, (blue)[DHAP]; **C:** (light-brown)[FBP], (dark-brown)[GAP]; **D:** (red) *J_CPK_*, after about 30 s this flux oscillates around zero, (dark-green) *J_MK_*; **E:** (orange) *J_SY_* plus *J_CAC_*, (black) *J_AE_*, (pink) *J_Pi_*; **F:** (dark-blue) dissipation function *Φ_W_* (Phi(W)), (light-blue) dissipation function *Φ_W_^p^* (Phi(ATPc)), (blue) power output *P_W_*^τ^ (Pload); SIM*_GlOx_*.

Up till now all results were related to steady state conditions, intrinsic oscillations do not occur under these conditions and forced oscillations were excluded. VMs in heart tissue, however, contract rhythmically at various frequencies and variable pressure developments. These oscillating contractions are known to be induced by rhythmic depolarizations of the membrane potential (*Δ_c_φ*), which in turn trigger periodic Ca^2+^ release from and accumulation into the sarcoplasmic reticulum (SR). The oscillating [Ca^2+^]_c_ then may initiate—beside other activations—periodic contraction and relaxation of myofibrils. Thus, such [Ca^2+^]_c_ oscillations are not generated by an intrinsic oscillator, but rather are forced by rhythmic depolarizations of *Δ_c_φ* It seems justified, therefore, to simulate rhythmic behavior by simple sinusoidal oscillations of [Ca^2+^]_c_ (A18). Figure 7 shows that many variables and parameters are forced to oscillate in the presence of [Ca^2+^]_c_ oscillations. The arithmetic means of such oscillations deviate slightly from steady state results. For instance, when results of Figure 6 are compared with results found under the same conditions, but in the presence of oscillations, they yield the following: at [Ca^2+^]_c_ = 0.18 μM *J*_O2_ and *P_Wτ_* are 0.121 μM/ms and 3.273 J/Ls, respectively, in the absence of oscillations. In the presence of forced oscillations, respective means are 0.122 μM/ms and 3.432 J/Ls. Values at 0.72 μM [Ca^2+^]_c_ are 0.570 μM/ms and 96.45 J/Ls, and 0.553 μM/ms and 95.18 J/Ls, respectively. Therefore, it seems justified to conclude that deductions made with respect to steady state are valid also under conditions of forced oscillations.

#### 2.3.3. Conductance Matching for Glucose Oxidation and Power Output

Because OP cannot be formulated as a two-flux-system, Equation (10o) would be inappropriate to show that power output of coupled glucose oxidation is near maximal. The problem of non-reliable overall parameters can be circumvented, however, if transformed conductances were taken into account.

It is well known in electrical engineering that power output of a battery becomes maximal when the internal conductance *L_i_* matches the conductance of the outer circuit, *L_e_*. A battery can be regarded as a coupled two-flux-system, in which a chemical reaction with affinity *A*_2_ is coupled to an electrical potential difference, *Δφ* = −*U*, with affinity *A*_1_. Coupled glucose oxidation, when transformed to equal flux velocities, can be regarded analogously: the input force is given by *A⃗_ov_*, while the coupled output force is given by −*A⃗_ATPc_*. Both forces and conductances *L⃗_i_* and *L⃗_e_* can be obtained from respective dissipation functions as described above. In analogy to a simple electric circuit consisting of a battery whose poles are connected by an electrical resistance (*R_e_* = 1/*L_e_*) with an attached load, *L⃗_i_* is related to the whole pathway of glucose oxidation including coupled ATP_c_ production, and *L_e_* to ATP_c_ utilizing reactions, *i.e.*, all cytosolic ATPases. As in an electric circuit, only the electrical resistance (*R_e_*) of an energy converter is involved with power maximation. In energy metabolism it is therefore only that part of *L⃗_W_*, which is associated with ATP splitting. That is, the flux through *A_ATPc_* (*J_W_^p^*) is the relevant flux, and *L_e_* therefore has to be derived from the input dissipation function *Φ_W_^p^*, and not from total dissipation function *Φ_W_* = *Φ_W_^ld^* + *Φ_W_^p^*. It is given by

(10a) $${\vec{L}}_{e}=\frac{\Phi_{W}^{p}}{{\vec{A}}_{ATPc}^{2}},\text{and }{\vec{A}}_{ATPc}=\frac{\Phi_{W}^{p}}{J_{GK}}$$

*L⃗_i_* is given by

(10b) $${\vec{L}}_{i}=\frac{\Phi_{i}}{{\vec{A}}_{i}^{2}},\text{with }\Phi_{i}=\Phi_{tot}-\Phi_{W},\text{and }{\vec{A}}_{i}=\frac{\Phi_{i}}{J_{GK}}$$

Delivery of free energy from coupled glucose oxidation in the form of ATP_c_, *i.e.*, power output, is identical to the input dissipation function of the load reaction

(10c) $$P_{ATPc}=\Phi_{W}^{p}=J_{GK}\;{\vec{A}}_{ATPc}$$

Analogous to $I=\frac{E}{R_{e}+R_{i}}$ (*I* = electrical current, and *E* = electromotive force), the respective flux is given by

(10d) $$J_{GK}=\frac{{\vec{A}}_{ov}}{\frac{1}{{\vec{L}}_{e}}+\frac{1}{{\vec{L}}_{i}}}$$

and

(10e) $${\vec{A}}_{ATPc}={\vec{A}}_{ov}-\frac{1}{{\vec{L}}_{i}}J_{GK}$$

Power output can be expressed then as

(10f) $$P_{ATPc}={\vec{A}}_{ov}^{2}\frac{1}{{\vec{L}}_{e}}\frac{1}{{\left(\frac{1}{{\vec{L}}_{e}}+\frac{1}{{\vec{L}}_{i}}\right)}^{2}}$$

or with $\Lambda =\frac{{\vec{L}}_{e}}{{\vec{L}}_{i}}$

(10g) $$P_{ATPc}={\vec{L}}_{i}{\vec{A}}_{ov}^{2}\frac{\Lambda }{{\left(1+\Lambda \right)}^{2}}$$

If *L⃗_i_* and *A⃗_ov_* were both constant, then *Φ_W_* *^p^* would be a function of *Λ* alone with a maximum at *Λ* = 1.0, or at *L⃗_e_* = *L⃗_i_*

With $\eta =-\frac{\left(-{\vec{A}}_{ATPc}\right)}{{\vec{A}}_{ov}}$, and using (10d) and (10f), efficiency in terms of *Λ* is given by

(10h) $$\eta =\frac{1}{1+\Lambda }$$

and *Λ* = 1, yields *η* = 0.5.

**Figure 8:**
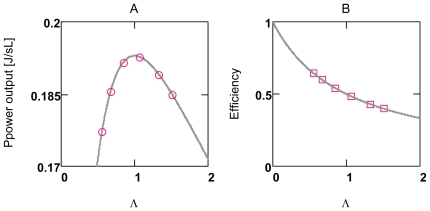
Power output and efficiency, respectively, *versus* conductance ratio *Λ* =*L⃗_e_* *L⃗_i_*. **A:** (red circles) results from a simulation with constant conductances, (line) analytical curve according to Equation (10g); **B:** (red squares) results from simulation, (line) analytical curve according to Equation (10h); SIM*_GLY_*.

These relations are verified with a simplified simulation (SIM*_GLY_*, A14) with constant conductances (Figure 8). The results show that a maximum does in fact exist at *Λ* = 1.0 under these conditions and that *η* is equal to 0.5 at this value of *Λ*.

**Figure 9:**
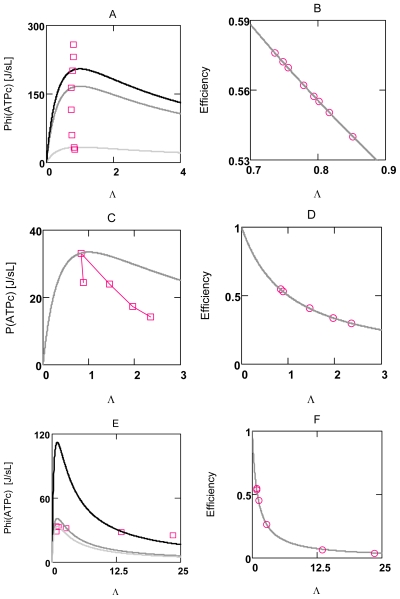
Power output and efficiency, respectively, *versus* conductance ratio *Λ* =*L⃗_e_* *L⃗_i_*. **A:** power output *P_ATPc_* =*Φ_W_^p^* (P(ATPc)) at various [Ca^2+^]_c_ activations of metabolism (in μM: 0.108, 0.18, 0.36, 0.54, 0.72, 1.08, 1.44, 1.80), (red squares) results from simulation, (lines, not all lines are shown) hypothetical curves according to (10g); **B:** efficiency at these activations, (red circles) results from simulation, (line) analytical curve according to (10h); **C** and **D:** as in **A** and **B**, but in the absence of [AMP]_c_ activation of PFK, (line, only the curve for 0.18 μM [Ca^2+^]_c_ is shown); **E** and **F:** as in **A** and **B**, but under uncoupled conditions. **E:** (light-grey line) uncoupling of *J* *_NA_* (*λ_n_^h^* = *λ_n_^o^* = 0.14 ), (grey line) uncoupling of *J* *_FA_* (*λ_f_^h^* = *λ_f_^o^* = 10), (black line) indirect uncoupling by increasing the proton leak conductance *J_PL_* of the inner membrane by a factor of 100, not all lines are shown; **F:** results from simulation fulfill the analytical curve according to (10h); SIM*_GlOx_* also under uncoupled conditions over a wide range.

A simulation of whole glucose oxidation with variable conductances (SIM*_GlOx_*, A15) and, in addition, with a feed back by [AMP]_c_ on PFK leads however to entirely different results, when *Λ* is increased by an increase of [Ca^2+^]_c_ (Figure 8) (conductances of load reactions become increased at an elevated [Ca^2+^]_c_, A4 and A5). Only one point at *Λ* = 0.82 ([Ca^2+^]_c_ = 0.18 μM) fulfils Equation (13g), all other values for *Φ_W_^p^*, and especially those found for higher [Ca^2+^]_c_’s are markedly elevated above that curve, but are all at nearly the same value of *Λ* (Figure 9A). In contrast, when PFK activation by [AMP]_c_ is omitted, power output cannot be increased by increasing *L⃗_e_* (Figure 9C) and, because conductances are not constant, points do not fulfill Equation (13g).

Obviously, the feedback by [AMP]_c_ on PFK can activate whole coupled glucose oxidation to appropriately increase ATP production to meet the demands of an increased ATP consumption. *Λ* remains remarkably constant at about 0.79 over a nine fold range of power output. It is close to maximal power output. Consequently, also efficiency must be approximately constant and, as follows from Equation (13g), near a value of 0.5 (0.56) (Figure 9B, D). Interestingly, also efficiency of the glycolytic span from glucose to pyruvate lies near this value.

The respective points of *Φ_W_^p^* must always fulfill the associated hypothetical power curve calculated each time for a given pair of values *L⃗_i_*; and *A⃗_ov_*. These curves are hypothetical insofar as especially *L⃗_i_* (changes of *A⃗_ov_* are negligible under all conditions) is not constant at varying *L⃗_e_*. That is, only one single point of the curve related to a given particular condition is of significance.

Larger deviations of *Λ* from 1.0 can be obtained by uncoupling OP. Figure 9F shows that efficiency obeys exactly Equation (13h) over a very wide range of *Λ*’s. Both conductances, *L⃗_e_* as well as *L⃗_i_*, are increased by uncoupling OP, but *L⃗_e_* to a much higher degree, so that *Λ* increases and thus efficiency decreases. Figure 9E shows that also under conditions of markedly increased *Λ*’s, power output points are close to their hypothetical respective power curve. Comparison of these uncoupled values with those obtained for coupled conditions shows that larger losses of useful power obviously can be prevented. This power preservation is associated, however, with a marked reduction in efficiency (Figure 9E,F).

These results demonstrate in a striking manner, how serious deteriorations of energy coupling can be compensated by an [AMP]_c_ mediated increase of pathway flux. In addition, for the metabolism of VMs they show that maintenance of power output obviously has priority over conservation of efficiency.

### 2.4. Replenishment of [ATP]_c_

In VMs there are three well known pathways of [ATP]_c_ replenishment: coupled glucose oxidation, which is described above, and two near equilibrium reactions catalyzed by creatine kinase (CK) and adenylate kinase (AK), respectively.

It is widely accepted that the primary tasks of the phosphocreatine (PCr) system is to buffer [ATP]_c_ and to shuttle it between mitochondria and cytosolic locations of high ATP usage like actomyosin ATPases in the myofilament lattice [48,49]. In addition, at least in present simulations, the PCr system is also interconnected with [AMP]_c_ dependent activation of PFK and thus, with activation of whole coupled glucose oxidation. The reduced [ADP]_c_ production through the action of the PCr system counteracts PFK activation. Therefore, delivery of free energy may depend also on this reaction.

While PCr shuttling is not included in simulations, AK and CK catalyzed reactions are present in all simulations of complete glucose oxidation. So, simulations without activation of PFK may show to what extent this activation may contribute to [ATP]_c_ replenishment and power output. In the absence of [AMP]_c_ activation of PFK and without PCr buffering, an increase of [Ca^2+^]_c_ from 0.18 to only 0.32 μM is sufficient to increase [ADP]_c_ from 80.8 μM to 4.24 mM (an increase of O_2_ consumption and *Φ_W_^p^* is abolished under these conditions). When PCr buffering can occur in the absence of [AMP]_c_ activation, [ADP]_c_ elevation is less pronounced (to 2.88 mM). On the other hand, under conditions of activation, an increase of [ADP]_c_ is markedly abolished (to 110.14 μM). Additional PCr buffering then is virtually without effect (108.75 μM).

Perhaps the most important task of the PCr system may be to function as a shuttle for [ATP^4−^] and [ADP^3−^]_c_ [26], for these compounds do not easily permeate the outer mitochondrial membrane. In addition, they must overcome diffusional restrictions inside the myofilament lattice, where myosin ATPases are located. Reaching of ATPases by diffusion at other locations, e.g., Na-K ATPases at the sarcolemma, or Ca^2+^ ATPases at the sarcoplasmic reticulum, may also be aggravated by diffusional restrictions. Since these ATPases are embedded in the filamentous network of the cytoskeleton, which likewise may be of limited accessibility for molecules like ionic ADP and ATP species. Supposedly such structures are charged at their interface between the cytosol and the network and may produce a Donnan potential, which, because of fixed negative net charges, must be negative inside the filament lattice. Because the shuttle molecules may be also charged, any shuttle mechanism has to take into account electrogenic crossing of the outer mitochondrial membrane on the one hand, and charged interfaces of myofibrils and/or cytoskeletons on the other hand. The following scheme describes the principals of PCr shuttling: starting with an increased ATP splitting in the myofilament lattice (‘f’) by an elevation of [Ca^2+^]_c_ leads to (in chemical notation)

(R1a) $${\text{MgATP}}_{\text{f}}^{2-}\to {\text{ADP}}_{\text{f}}^{3-}+{\text{H}}_{2}{\text{PO}}_{4\text{f}}^{-}+{\text{Mg}}_{\text{f}}^{2+}$$

(R1b) $${\text{ADP}}_{\text{f}}^{3-}+{\text{PCr}}_{\text{f}}^{2-}+{\text{H}}_{\text{f}}^{+}⇌{\text{ATP}}_{\text{f}}^{4-}+{\text{Cr}}_{\text{f}}$$

and

(R1c) $${\text{ATP}}_{\text{f}}^{4-}+{\text{Mg}}_{\text{f}}^{2+}⇌{\text{MgATP}}_{\text{f}}^{2-}$$

In the filament compartment MgATP^2−^ is recycled, whereas PCr^2−^ and Cr are consumed and produced, respectively. In the intermembrane compartment similar reactions occur, but with opposite directions. From the adenine nucleotide exchange reaction (*J*_AE_) at the inner membrane, ATP^4−^ is delivered to the intermembrane space (‘im’), and ADP^3−^ is transported into the matrix. Both are recycled by the PCr reaction in this compartment,

(R1d) $${\text{ATP}}_{\text{im}}^{4-}+{\text{Cr}}_{\text{im}}⇌{\text{ADP}}_{\text{im}}^{3-}+{\text{PCr}}_{\text{im}}^{2-}+{\text{H}}_{\text{im}}^{+}$$

Because of the limited permeability of the VDAC pore for adenine nucleotides, [ATP^4−^]_im_ and [ADP^3−^]_im_ cannot be equilibrated rapidly with the cytosol. The associated affinity, *A_ATPim_*, may be high, so that here the PCr_im_ reaction is forced into the direction shown in reaction (R1d). In contrast, in the environment of ATPases, *A_ATPf_* may be lower, whereby here the PCr_f_ reaction may be forced into the reverse direction ((R1a)). At both locations the PCr system may be near equilibrium, but because of different *A_ATP_*’s, at different respective mass action ratios in both compartments. Two opposed concentration gradients, one of [PCr^2−^] and one of [Cr] are produced, driving diffusion of PCr^2−^ from mitochondria to myofilaments, and Cr from myofilaments to mitochondria. The crossing of the outer mitochondrial membrane and the myofilament/cytosol interface by PCr^2−^ occurs via electroneutral exchange against HPO_4_^2−^. In the myofilament compartment, one H^+^ from H_2_PO_4_^−^ restores one consumed proton by the PCr reaction. A third concentration gradient formed by [HPO_4_^2−^] drives diffusion of this ion from myofilaments to mitochondria. In the intermembrane space, HPO_4_^2−^ takes up one H^+^ produced here from the reversed PCr reaction and finally enters as H_2_PO_4_^−^ ion H/Pi symport at the inner membrane.

As a result ATP, is transported from mitochondria to ATPases and in its split form back to mitochondria. As a prerequisite, in addition to the two-compartment system of classical cellular energetics, a third compartment for *A_ATP_*, the mitochondrial intermembrane space is necessary to guarantee gradient formation for PCr shuttling and a high power output. As a result, however, a certain amount of free energy of ATP production becomes inevitably dissipated by the diffusional flows along these gradients.

During repetitive contraction cycles oscillations of respective flows can be expected (Figure 1). It is questionable, however, that [ATP]_c_ and [PCr] oscillations occurred with such high peak values of about 10% as was found by Honda *et al*. [50], as this would require extreme rates of O_2_ consumption during recovery phases. When the metabolism of VMs is changing from a low to a high power output, the rate of ATP_c_ consumption becomes elevated above the rate of ATP_c_ production, so that *A_ATPc_* is lowered, and the PCr reaction begins to partially compensate the decline of *A_ATPc_*. After a certain delay, consumption and production rates match again, and *A_ATPc_* is adjusted to a new lowered value, which forces the corresponding near equilibrium PCr reaction to a new mass action ratio with a lower [PCr]. *A_CPK_* oscillates now again around zero. When power output switches back to lower values, the reverse processes lead to a re-increase of *A_ATPc_* until respective ATP_c_ rates match. The PCr reaction again oscillates around zero driving force, but now at a re-increased [PCr]. Supposedly, it is not necessary that concentrations of the PCr system must be changed during a contraction cycle to allow formation of sufficiently steep gradients. As mentioned above, this might be brought about by compartmentalization of *A_ATPc_*.

Gradients of [PCr^2−^] and [Cr] may be produced preferably at interfaces of mitochondria/cytosol and myofibrils/cytosol, respectively. For the maintenance of flows, it is necessary that compartments of high and low *A_ATP_* are in close proximity, because otherwise gradients could be destroyed by soluble CK of the cytosol which may be present also in this compartment at high activity. Such structural prerequisites obviously are realized in VMs, in which mitochondria surround myofibrils and the sarcolemma in a highly ordered way.

### 2.5. Pancreatic β-Cells

The primary task of β-cells is to secrete insulin in response to an elevated glucose concentration. The process of secretion involves exocytosis of insulin containing vesicles, which are [Ca^2+^]_c_ and [ATP]_c_ dependent like other load reactions of the cytosol. The sequence of events, however, leading to an activating increase of [Ca^2+^]_c_ in β-cells is remarkably different from activation of VMs. In contrast to this latter cell type, an increase of [Ca^2+^]_c_ in the β-cell is induced intracellularly by an increase of pathway fluxes of coupled glucose oxidation and associated decrease of [ADP]_c_ (increase of *A*_ATPc_). The lowered [ADP]_c_ in turn closes ATP-sensitive K^+^ channels (K_ATP_), leading to a depolarization of *Δ_c_* *φ* and an influx of Ca^2+^ from the extracellular space through opened (by depolarization) voltagegated Ca^2+^ channels (Ca_V_) into the cytosol [51]. Such a metabolically induced mechanism of [Ca^2+^]_c_ increase can certainly only be realized by oscillations. A low [ADP]_c_ and an elevated [Ca^2+^]_c_ cannot coexist over a longer period, because an increased [Ca^2+^]_c_ would activate cytosolic load reactions including insulin exocytosis, which in turn leads to a re-increase of [ADP]_c_, a repolarization of *Δ_c_φ* via opening of K_ATP_ channels, and a re-closure of Ca_V_ channels. A metastable state must be generated, which is characterized by oscillations with a phase shift between the two variables [Ca^2+^]_c_ and [ADP]_c_ [6,10]. That is to say, the question why stimulus-secretion coupling of the β-cell must be associated with oscillations is explained by the metabolically induced reaction sequence, which itself represents the intrinsic part of an oscillator.

The initiation of an increased glucose metabolism, in contrast to VMs, does not proceed via activation of PFK by [AMP]_c_, but through activation of glucokinase by cytosolic [Glu]. Glucokinase has a much higher K_M_ value than hexokinase from heart muscle and, therefore, is not saturated at resting (4.0–5.0 mM) [Glu]s. So, an increase of [Glu] from resting levels (4.0 mM) to 10.0 mM, although without an effect in VMs, activates glucose metabolism in β-cells. An increase of [Ca^2+^]_c_ and [AMP]_c_ is not needed for this kind of activation. The increase of [Glu] acts as a stimulus for insulin secretion, whereas the glucokinase reaction, beside its function as a hexokinase in GLY, constitutes in addition the recognition mechanism for this special stimulus.

Glucokinase of β-cells is known to be not inhibited like hexokinase by [G6P]. In simulations of glucose metabolism of VMs, the inhibition by the latter variable is needed to make this first reaction step more flexible for the adjustment of a steady state. Because [Glu] is not a variable but a fixed parameter, flexibility of the first step of GLY in β-cells has been introduced by contracting the first three reactions of GLY, including the PFK reaction to one single step (see [6]). Now the first step of GLY in simulations possesses an activation factor containing [ADP]_c_ as a variable, so that certain flexibility also for β-cell simulations may be ensured. This is the main difference (beside different geometrical parameters and conductances) between simulations of whole coupled glucose oxidation of VMs and β-cells. In these latter cells the concentration of total adenine nucleotide concentrations is, however, much lower than in VMs. In β-cells, especially [ATP]_c_ is much lower (in simulations 3,740 μM compared to 8,820 μM). The responsiveness of PFK and PK to the inhibitory action of [ATP]_c_, therefore, may be drastically lowered in β-cells, even if in this cell type the same isoenzymes as in VMs were expressed. That is, the functional characteristics of these enzymes might be markedly changed simply by a lowering of [ATP]_c_.

The above described mode of glucose recognition has its price, however. Compared with VMs, delivery of free energy in β-cells via coupled glucose oxidation is drastically cut back through the limited capability of GK to respond on [Glu] activation. Power output at 4.0 mM [Glu] can be increased only up to a [Ca^2+^]_c_ of about 0.54 μM. This corresponds to a factor of only about 1.5 in β-cells compared to a factor of about 9.0 in VMs. Also the tolerance toward uncoupling is extremely reduced in β-cells. Uncoupling of ATP synthase already at *λ_sp_* = *λ_sh_* = 0.03 (*q*_s_ = 0.97) causes a reduction of power output (0.18 μM [Ca^2+^]_c_) in these cells of about 43 %, and an increase of [ADP]_c_ from 260 to 3153 μM. At higher [Ca^2+^]_c_’s, these effects are even more pronounced.

During periods of hypoglycaemia, β-cells would be even more easily affected. To counteract such a risk, it is indispensable that delivery of free energy can be partly provided by a parallel fuelling of other substrates like fatty acids and/or amino acids into energy metabolism.

At a [Glu] of 10.0 mM β-cells are less susceptible to cell damage, but insulin release can be seriously jeopardized by a relative weak uncoupling. Under activated conditions at very low values of *λ_sp_* = *λ_sh_* = 0.015, oscillations disappear and insulin exocytosis is decreased to 7.6% (not shown).

From this behavior of β-cells, a fundamental conclusion with respect to the degree of coupling of mitochondrial inner membrane reactions can be reached: especially ATP synthase must be tightly coupled, because otherwise [ADP]_c_ in β-cells would be markedly increased. Furthermore, because it seems plausible to suggest that mitochondria of other cell types like muscle cells might not differ in this respect, a tight coupling of inner mitochondrial membranes can be expected.

**Figure 10:**
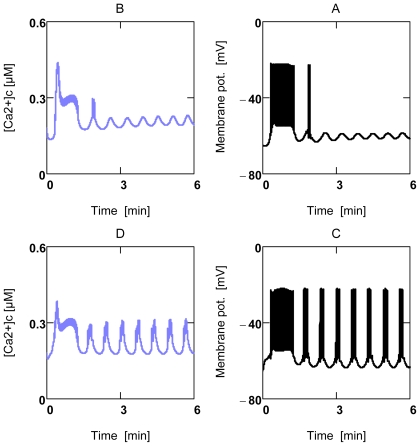
Effect of the PCr system in β-cells. *Δ_c_φ* (**A** and **C**) and [Ca^2+^]_c_ (**B** and **D**) in the presence of the PCr system, [Cr_tot_] = 2.0 mM (**A** and **B)**, [Cr_tot_] = 0,5 mM (**C** and **D**)). Fluxes *J_AK_* and *J_CPK_* (A10 and A11) are added to a simulation taken from reference [10].

As is already described above, the PCr system shuttles ATP free energy between mitochondria and ATPases. It is questionable, however, if this system of facilitated transport may be operative in β-cells, because the buffering effect of the PCr system might interfere with the oscillatory machinery of stimulus-secretion coupling. Figure 10 shows results from a β-cell simulation (taken from [10]), in which the PCr system additionally has been included. Stimulation of β-cell activity by 10.0 mM [Glu] is possible only if [PCr_tot_] does not exceed 1.0 mM. At higher concentrations normal oscillations of *Δ* *_c_φ* and [Ca^2+^]_c_ cannot be elicited anymore, whereby insulin release becomes markedly reduced. In the absence of PCr shuttling, normal functioning of stimulus-secretion coupling nevertheless may be possible, since the oscillatory frequency of activated β-cells is very low compared to rhythmic contractions of VMs, so that diffusional restrictions for adenine nucleotides cannot impair power output.

## 3. Methods

For a treatment of coupled glucose oxidation as an integral part of cellular energetics, cell membrane and SR reactions are of secondary importance. Therefore, to simplify expressions, all reactions of the cell membrane and *Δ* *_c_φ* are omitted in present simulations. In addition, also most of SR reactions are excluded, only Ca^2+^ pumping of the sarco/endoplasmatic reticulum Ca^2+^ ATPase (SERCA) as an ATP consuming reaction is included in simulations. [Ca^2+^]_c_ therefore appears in calculations not as a variable but as an adjustable parameter.

Because of different geometrical parameters, volume fractions and *α* values have to be adapted for VMs. Data from Aliev *et al*. [52] are used to calculate volumes of cellular compartments. A value of 50 and 20% of *V_Cell_* (*V_Cell_* = 34.4 pL, [53]) is used for sarcosolic volume *V_c_*, and for total mitochondrial matrix volume *V_m_*, respectively. Capacitances of all mitochondrial inner membranes of 3.6 nF and 18.5 pF are used for VMs and β-cells, respectively. In addition, altered *α* values are obtained with *α_c_* = 0.6025759 10^12^, and *α_m_* = 1.50644 10^12^ μM/C, yielding a flux *J* = *α* × I 10^−18^ μM/ms (C = Coulomb, I = numerical value of current *I* in fA). From *α_m_*/*α* *_c_* = *V_c_*/*V_m_* a Q_Vm_ = *V_c_*/*V_m_* = 2.5 is obtained. This value is remarkably larger in β-cells (*Q_Vb_* = 14.4).

All other constants, especially those for [H^+^] and [Mg^2+^] dependent ATP hydrolysis and mitochondrial transport reactions are taken from reference [10]. Sarcosolic and mitochondrial pH values (pH_c_, pH_m_) of VMs are set to 7.1 and 7.4, in β-cells corresponding values are 7.2 and 7.5, respectively. [Mg^2+^]_c_ and [Mg^2+^]_m_ are set for both cell types to 0.8 mM. Constants for the AK and CK reaction (A10 and A11), respectively, are taken from Golding *et al*. [54].

Calculations were performed using Mathcad^®^ 14.0 M011 using Mathcad^®^ solvers *Radau* or *AdamsBDF*. Identical results were obtained from different program versions and/or solvers. Programs were run under Microsoft^®^ Windows XP Professional.

## 4. Conclusions

Applied equations are structurally similar to other phenomenological relations and especially to equations used in NET. But in contrast to these latter equations, the new equations contain variable conductances, which allow modeling of individual fluxes in analogy to the kinetic behavior known from enzyme-catalyzed reactions. At steady state, all variables are constant, so that flux equations presented here become undistinguishable from those of NET and Michaelis-Menten type equations. The great advantage over the latter rate laws, however, is given by its remarkable stability towards perturbations and its ability to reach a new steady state after such a perturbation in a possible short interval. This behavior of equations makes them suitable for modeling studies of metabolic networks and, in addition, for elucidation of pathway control mechanisms.

In simulations of coupled glucose oxidation, a drastic increase of free energy flow to increase power output can be achieved only at two points, at [Ca^2+^]_c_ dependent load reactions and at PFK. For maximal power output respective conductances of in—and output fluxes must be similar in magnitude. This conductance matching is sufficiently fulfilled over a wide range of useful power generation. It may represent the basic mechanism of energy metabolism to ensure a high cellular power output under conditions of a sufficient supply of substrate and oxygen. Moreover, it may be regarded as a necessary prerequisite for the development of so-called fight or flight behavior, as it can be observed in higher vertebrates as well in more primitive forms.

In β-cells, the GK enzyme is limiting the metabolic rate of coupled glucose oxidation, whereby the metabolic mechanism of glucose recognition is made possible. Obviously, only a few changes in the first reaction steps of glucose metabolism are sufficient to produce that remarkably large difference of power output between VMs and β-cells. On the other hand, both cell types differ enormously not only in size and geometry, but also in their markedly different degrees of structural organization. This strongly indicates that a task such as high power output may be guaranteed only if, in addition to adequately controlled pathways, that appropriate structural realities also exist. Only in this way, conductance matching for a maximal power output can be realized.

## Appendix

### 1. Ventricular Muscle Cells

Nearly all individual fluxes of coupled glucose oxidation are given in references [6] and [10]. In VMs only the PFK reaction is added to these pathway fluxes, leading also to changes of the formerly more contracted flux through GK plus PFK. The flux through GK for VMs contains [F6P] inhibition and is given by

A1 $$J_{GK}=\frac{{\left[\text{Glu}\right]}^{2}}{{\left(K_{M}^{Hex}\right)}^{2}+{\left[\text{Glu}\right]}^{2}}\frac{1}{1+e^{\left(\frac{\left[\text{F}6\text{P}]-[\text{F}6{\text{P}}_{05}\right]}{\left[{\text{S}}_{\text{F}6\text{P}}\right]}\right)}}L_{Gkm}^{\text{max}}\times RT\text{ln}\left(\frac{\left[\text{Glu}\right]}{\left[\text{F}6\text{P}\right]}\frac{{\left[\text{ATP}\right]}_{\text{c}}}{{\left[\text{ADP}\right]}_{\text{c}}}\times K_{GK}^{\prime }\right)$$

[ATP]_c_ = [A_tot_] − [ADP]_c_ −[AMP], A_tot_ = 9,000.67 μM, *K**_M_**^Hex^* = 100 μM, [F6P_05_] = 250 μM, [S_F6P_] = 20 μM, *L**_GKm_*^max^ = 2904 × 10^−8^ (μM/ms) × (mol/J), *K**_GK_**′* = 5.95 × 10^3^.

The PFK reaction is activated by [F6P], [AMP], and inhibited by [ATP]_c_. It is given by

A2 $$J_{PFK}=\frac{\left[\text{F}6\text{P}\right]}{\left(K_{M}^{F6P})+[\text{F}6\text{P}\right]}\frac{{\left[\text{AMP}\right]}^{2}}{{\left(K_{M}^{PFK}\right)}^{2}+{\left[\text{AMP}\right]}^{2}}\frac{1}{1+e^{\left(\frac{{\left[\text{ATP}\right]}_{\text{c}}-\left[{\text{ATP}}_{05}^{\text{PFK}}\right]}{\left[S_{\text{ATP}}^{\text{PFK}}\right]}\right)}}L_{PFK}^{\text{max}}\times RT\text{ln}\left(\frac{\left[\text{F}6\text{P}\right]}{\left[\text{FBP}\right]}\frac{{\left[\text{ATP}\right]}_{\text{c}}}{{\left[\text{ADP}\right]}_{\text{c}}}\times K_{PFK}^{\prime }\right)$$

*K**_M_**^F^* ^6^*^P^* = 300.0 μM, *K**_M_**^PFK^* = 2.0 μM, [ATP_05_^PFK^]= 8930, [S_ATP_^PFK^] = 20 μM, *L**_PFK_*^max^ = 1188 × 10^−8^ (μM/ms) × (mol/J), *K**_PFK_**′* = 1.348 × 10^4^.

For *J**_Ga_* of VMs inhibition of PK by [ATP]_c_ is included, yielding

A3 $$J_{Ga}=\frac{\left[\text{GAP}\right]}{K_{M}^{Ga}+[\text{GAP}]}\frac{1}{1+e^{\left(\frac{{\left[\text{ATP}\right]}_{\text{c}}-[{\text{ATP}}_{05}^{\text{Ga}}]}{\left[{\text{S}}_{\text{ATP}}^{\text{Ga}}\right]}\right)}}L_{Ga}^{\text{max}}\times RT\text{ln}\left(\frac{\left[\text{GAP}\right]}{{\left[\text{Pyr}\right]}_{\text{c}}}\times \frac{{\left[\text{ADP}\right]}_{\text{c}}^{2}{\left[\text{Pi}\right]}_{\text{c}}}{{\left[\text{ATP}\right]}_{\text{c}}^{2}[{\text{c}}^{0}]}\times \frac{{\left[{\text{NAD}}_{\text{ox}}\right]}_{\text{c}}}{{\left[{\text{NAD}}_{\text{red}}\right]}_{\text{c}}}\times K_{Ga}^{\prime }\right)$$

[*ATP*_05_^Ga^] = 8,900 μM, [*S*_ATP_^Ga^] = 10 μM, *L*_max_*^Ga^* = 7,392 × 10^−8^ (μM/ms) × (mol/J).

Load reactions are given by

A4 $$J_{Wser}=\alpha_{c}\times \frac{{\left[{\text{Ca}}^{2+}\right]}_{\text{c}}^{2}}{{\left(K_{M}^{Wser}\right)}^{2}+{\left[{\text{Ca}}^{2+}\right]}_{\text{c}}^{2}}G_{Wser}^{\text{max}}\times \left((\lambda_{Wserca}+1)\times \frac{RT}{F}\text{ln}\left(\frac{{\left[{\text{Ca}}^{2+}\right]}_{\text{c}}^{2}{\left[{\text{H}}^{+}\right]}_{\text{r}}^{4}}{{\left[{\text{Ca}}^{2+}\right]}_{\text{r}}^{2}{\left[{\text{H}}^{+}\right]}_{\text{c}}^{4}}+{\tilde{A}}_{ATPc}\right)\right)$$

A5 JWcrbr=11+exp([Ca2+]c-[Ca05Wcrbr][SCaWcrbr])LWcrbrmax×[-([Ca2+]c3(KMcrbr)3+[Ca2+]c3(KIcrbr)2(KIcrbr)2+[Ca2+]c2×ϕa+ϕb)+AATPc]

and

A6 $$J_{Wb}=L_{Wb}^{\text{max}}\times [RT\text{ln}(K_{Ld}^{Wb})+A_{ATPc}]$$

*K**_M_**^Wser^* = 0.4 μM, *G**_Wser_*^max^ = 600 pS, *λ**_Wser_* = 0, [H^+^]_r_ = [H^+^]_c_ = 10^−1.1^ μM, Ca_05_*^Wcrbr^* = 0.55 μM, S*_Ca_**^Wcrbr^* = 0.1 μM, *L**_Wcrbr_* ^max^ = 10^−8^ (μM/ms) × (mol/J), *K**_M_**^crbr^* = 0.4 μM, *K**_I_**^crbr^* = 1.5 μM, *φ**_a_* = 3 × 10^4^ J/mol, *φ**_b_* = 2 × 10^4^ J/mol, *K**_Ld_**^Wb^* = 10^−6^.

Fluxes of OP include now the possibility for uncoupling. They are given by

A7a $$J_{NA}^{h}=\frac{\alpha_{m}}{\nu_{n}}\frac{{\left[{\text{NAD}}_{\text{red}}\right]}_{\text{m}}^{2}}{{\left(K_{M}^{NA}\right)}^{3}+{\left[{\text{NAD}}_{\text{red}}\right]}_{\text{m}}^{3}}G_{NA}^{\text{max}}\times \left((\lambda_{nh}+1)\nu_{n}\frac{RT}{F}\text{ln}\left(\frac{{\left[{\text{H}}^{+}\right]}_{\text{m}}}{{\left[{\text{H}}^{+}\right]}_{\text{c}}}\right)+\frac{RT}{F}\text{ln}\left(\frac{{\left[{\text{NAD}}_{\text{red}}\right]}_{\text{m}}}{{\left[{\text{NAD}}_{\text{ox}}\right]}_{\text{m}}}\times \frac{{\left[{\text{O}}_{2}\right]}^{0.5}}{{\left[{\text{c}}^{0}\right]}^{0.5}}\times K_{NA}^{\prime }\right)\right),$$

and

A7b $$J_{NA}^{o}=\frac{\alpha_{m}}{\nu_{n}}\frac{{\left[{\text{NAD}}_{\text{red}}\right]}_{\text{m}}^{3}}{{\left(K_{M}^{NA}\right)}^{3}+{\left[{\text{NAD}}_{\text{red}}\right]}_{\text{m}}^{3}}G_{NA}^{\text{max}}\times \left(\nu_{n}\left(\frac{RT}{F}\text{ln}\left(\frac{{\left[{\text{H}}^{+}\right]}_{\text{m}}}{{\left[{\text{H}}^{+}\right]}_{\text{c}}}\right)+\Delta_{m}\varphi \right)+(\lambda_{no}+1)\frac{RT}{F}\text{ln}\left(\frac{{\left[{\text{NAD}}_{\text{red}}\right]}_{\text{m}}}{{\left[{\text{NAD}}_{\text{ox}}\right]}_{\text{m}}}\times \frac{{\left[{\text{O}}_{2}\right]}^{0.5}}{{\left[{\text{c}}^{0}\right]}^{0.5}}\times K_{NA}^{\prime }\right)\right)$$

*G**_NA_*^max^ = 3.306 × 10^5^ pS.

A8a $$J_{FA}^{h}=\frac{\alpha_{m}}{\nu_{f}}\frac{{\left[{\text{FAD}}_{\text{red}}\right]}_{\text{m}}^{2}}{{\left(K_{M}^{FA}\right)}^{3}+{\left[{\text{FAD}}_{\text{red}}\right]}_{\text{m}}^{3}}G_{FA}^{\text{max}}\times \left((\lambda_{fh}+1)\nu_{f}\left(\frac{RT}{F}\text{ln}\left(\frac{{\left[{\text{H}}^{+}\right]}_{\text{m}}}{{\left[{\text{H}}^{+}\right]}_{\text{c}}}\right)+\Delta_{m}\varphi \right)+\frac{RT}{F}\text{ln}\left(\frac{{\left[{\text{FAD}}_{\text{red}}\right]}_{\text{m}}}{{\left[{\text{FAD}}_{\text{ox}}\right]}_{\text{m}}}\times \frac{{\left[{\text{O}}_{2}\right]}^{0.5}}{{\left[{\text{c}}^{0}\right]}^{0.5}}\times K_{FA}^{\prime }\right)\right),$$

A8b $$J_{FA}^{o}=\frac{\alpha_{m}}{\nu_{f}}\frac{{\left[{\text{FAD}}_{\text{red}}\right]}_{\text{m}}^{2}}{{\left(K_{M}^{FA}\right)}^{3}+{\left[{\text{FAD}}_{\text{red}}\right]}_{\text{m}}^{3}}G_{FA}^{\text{max}}\times \left(\nu_{f}\left(\frac{RT}{F}\text{ln}\left(\frac{{\left[{\text{H}}^{+}\right]}_{\text{m}}}{{\left[{\text{H}}^{+}\right]}_{\text{c}}}\right)+\Delta_{m}\varphi \right)+(\lambda_{fo}+1)\frac{RT}{F}\text{ln}\left(\frac{{\left[{\text{FAD}}_{\text{red}}\right]}_{\text{m}}}{{\left[{\text{FAD}}_{\text{ox}}\right]}_{\text{m}}}\times \frac{{\left[{\text{O}}_{2}\right]}^{0.5}}{{\left[{\text{c}}^{0}\right]}^{0.5}}\times K_{FA}^{\prime }\right)\right)$$

*G**_FA_*^max^ = 4.959 × 10^5^ pS.

A9a $$J_{SY}^{p}=\alpha_{m}\cdot \nu_{s}\cdot \frac{{\left[\text{ADP}\right]}_{\text{m}}^{2}}{{\left(K_{M}^{SY}\right)}^{2}+{\left[\text{ADP}\right]}_{\text{m}}^{2}}\frac{{\left[{\text{Ca}}^{2+}\right]}_{\text{m}}^{2}}{{\left(K_{M}^{SY}\right)}^{2}+{\left[{\text{Ca}}^{2+}\right]}_{\text{m}}^{2}}G_{SY}^{\text{max}}\times \left((\lambda_{sp}+1)(-{\tilde{A}}_{ATPm})+\frac{1}{\nu_{s}}\left(\frac{RT}{F}\text{ln}\left(\frac{{\left[{\text{H}}^{+}\right]}_{\text{c}}}{{\left[{\text{H}}^{+}\right]}_{\text{m}}}\right)-\Delta_{m}\varphi \right)\right)$$

and

A9b $$J_{SY}^{h}=\alpha_{m}\cdot \nu_{s}\cdot \frac{{\left[\text{ADP}\right]}_{\text{m}}^{2}}{{\left(K_{M}^{SY}\right)}^{2}+{\left[\text{ADP}\right]}_{\text{m}}^{2}}\frac{{\left[{\text{Ca}}^{2+}\right]}_{\text{m}}^{2}}{{\left(K_{M}^{SYc}\right)}^{2}+{\left[{\text{Ca}}^{2+}\right]}_{\text{m}}^{2}}G_{SY}^{\text{max}}\times \left((-{\tilde{A}}_{ATPm})+(\lambda_{sh}+1)\frac{1}{\nu_{s}}\left(\frac{RT}{F}\text{ln}\left(\frac{{\left[{\text{H}}^{+}\right]}_{\text{c}}}{{\left[{\text{H}}^{+}\right]}_{\text{m}}}\right)-\Delta_{m}\varphi \right)\right)$$

For VMs [Ca^2+^]_m_ activation of ATP synthase is included with *K**_M_**^SYc^* = 0.5 μM, *G**_SY_*^max^ = 6.1161 × 10^5^ pS. Especially simulations of VMs contain both, AK catalyzed AMP production, and CK catalyzed ATP formation. Flux through AK is given by

A10 $$J_{AK}=\frac{{\left[\text{ADP}\right]}^{2}}{{\left(K_{M}^{MK}\right)}^{2}+{\left[\text{ADP}\right]}^{2}}L_{AK}^{\text{max}}\times RT\text{ln}\left(\frac{{\left[\text{ADP}\right]}^{2}}{\left[\text{AMP}][[{\text{A}}_{\text{tot}}]-[\text{ADP}]-[\text{AMP}]\right]}K_{AK}^{ref}\frac{P_{ATP4-}\;P_{ATP2-}}{P_{ADP3-}^{2}}\right)$$

and flux through CK by

A11 $$J_{CK}=\frac{{\left[\text{ADP}\right]}^{2}}{{\left(K_{M}^{CPK}\right)}^{2}+{\left[\text{ADP}\right]}^{2}}L_{CK}^{\text{max}}\times RT\text{ln}\left(\frac{\left[\text{ADP}][\text{PCr}\right]}{\left[[{\text{A}}_{\text{tot}}]-[\text{ADP}]-[\text{AMP}]][[{\text{Cr}}_{\text{tot}}]-[\text{Pcr}]\right]}\frac{{\left[{\text{H}}^{+}\right]}_{c}}{\left[{\text{c}}^{0}\right]}K_{CK}^{ref}\frac{P_{ATP4-}}{P_{ADP3-}\;P_{PCr2-}}\right)$$

*L**_AK_*^max^ = 30 × 10^−8^ (μM/ms) × (mol/J), *K**_AK_**^ref^* = 0.374, *K**_M_**^AK^* = 30.0 μM. *L**_CK_*^max^ = 36 × 10^−5^ (μM/ms) × (mol/J), *K**_CK_**^ref^* = 3.77 × 10^8^, *K**_M_**^CK^* = 30.0 μM. The following binding polynomials are used:

A12a $$P_{AMP2-}=1+\frac{\left[{\text{H}}^{+}\right]}{K1am}+\frac{\left[{\text{Mg}}^{2+}\right]}{K2am}$$

and

A12b $$P_{PCr2-}=1+\frac{\left[{\text{H}}^{+}\right]}{K1PCr}+\frac{\left[{\text{Mg}}^{2+}\right]}{K2PCr}$$

The above flux equations show that often several activating or inhibiting factors may be necessary to model a given flux. Principally these probability factors can be replaced by one single factor (=*v V**_Enz_*^max^, *v* = *J**_M-M_*) obtained from the given rate law of enzyme kinetics.

### 2. Oxidative Phosphorylation

The fraction *Q**_H_* of input fluxes *J**_NA_* plus *J**_FA_* for OP is given by

A13a $$Q_{H}=\frac{\nu_{n}J_{NA}^{h}+\nu_{f}J_{FA}^{h}-(J_{Ms}+J_{PL}+2J_{HCE}+J_{CAC})}{\nu_{n}J_{NA}^{h}+\nu_{f}J_{FA}^{h}}=\frac{\frac{1}{\nu_{s}}J_{SY}^{h}+(J_{AE}-J_{CAC})}{\nu_{n}J_{NA}^{h}+\nu_{f}J_{FA}^{h}}$$

Fluxes, affinities, and conductances for OP can be obtained from respective dissipation functions yielding

A13b $$\Phi_{Rop}^{o}+\Phi_{Rop}^{h}=\frac{Q_{H}}{QV}(J_{R}^{o}A_{R}^{o}+J_{R}^{h}A_{R}^{h})$$

with *J**_R_* = *J* *_NA_* + *J**_FA_*. *A**_R_* denotes the associated affinity of this flux.

The dissipation function for OP plus an attached load reaction (ATP cycling) is given by

A13a $$\Phi_{OPL}=J_{Rop}^{o}A_{R}^{o}+J_{Rop}^{h}A_{R}^{h}+J_{SY}^{h}A_{SY}^{h}+(J_{AE}-J_{CAC})\Delta {\tilde{\mu }}_{H}+J_{SY}^{p}A_{ATPm}+(J_{AE}-J_{CAC})(A_{AE}+A_{pi})+J_{Wop}^{p}A_{W}^{p}+J_{Wop}^{ld}A_{W}^{ld}$$

The terms

A13d $$J_{Rop}^{h}A_{R}^{h}+J_{SY}^{h}A_{SY}^{h}+(J_{AE}-J_{CAC})\Delta {\tilde{\mu }}_{H}=0$$

describe proton cycling; their sum vanishes at steady state. The following three terms participate in ATP cycling, also this sum is equal to zero at steady state.

A13e $$J_{SY}^{p}A_{ATPm}+(J_{AE}-J_{CAC})(A_{AE}+A_{Pi})+J_{Wop}^{p}A_{W}^{p}=0$$

The following equations must be fulfilled: for proton cycling

A13f $$\nu_{Rop}J_{Rop}^{h}=\frac{J_{Sy}^{h}}{\nu_{s}}+(J_{AE}-J_{CAC})$$

and for ATP cycling

A13g $$A_{ATPm}+A_{AE}+A_{Pi}=-A_{ATPc}=-A_{W}^{p}$$

A13h $$\frac{J_{SY}^{p}}{QV}=\frac{J_{AE}-J_{CAC}}{QV}=J_{Wop}^{p}$$

### 3. Simulations

SIM_GLY_:

A14 $$\begin{array}{l} \frac{d[\text{FBP}]}{dt}=J_{GK}-J_{Al}\\ \frac{d[\text{GAP}]}{dt}=J_{Al}+J_{Ti}-J_{Ga}\\ \frac{d[\text{DHAP}]}{dt}=J_{Al}-J_{Ti}\\ \frac{d{\left[\text{ADP}\right]}_{\text{c}}}{dt}=J_{W}+2J_{GK}-2J_{Ga}\\ \frac{d{\left[\text{Pi}\right]}_{\text{c}}}{dt}=J_{W}-J_{Ga} \end{array}$$

*J*_W_ is overtaken from [6]. *L**_W_*^max^ is changed to 99 × 10^−8^ (μM/ms) × (mol/J).

SIM_GlOx_:

A15 $$\begin{array}{l} \frac{d(\Delta_{m}\varphi )}{dt}=\frac{1}{{Cm}_{m}\cdot \alpha_{m}}\times \left(\frac{1}{\nu_{s}}J_{SY}+J_{AE}+2J_{CU}+J_{MS}+J_{PL}-\nu_{n}J_{NA}-\nu_{f}J_{FA}\right)\\ \frac{d{\left[{\text{H}}^{+}\right]}_{\text{m}}}{dt}=\frac{1}{1+\kappa_{1}^{BU}+\kappa_{2}^{BU}}\times \left(\frac{1}{\nu_{s}}J_{SY}+J_{Pi}+J_{PL}+J_{MS}+2J_{HC}+J_{CAm}-\nu_{n}J_{NA}-\nu_{f}J_{FA}\right)\\ \frac{d{\left[{\text{NAD}}_{\text{red}}\right]}_{\text{m}}}{dt}=J_{PDH}+3J_{CAC}+J_{MS}-J_{NA}\\ \frac{d{\left[{\text{FAD}}_{\text{red}}\right]}_{\text{m}}}{dt}=J_{CAC}+J_{GS}-J_{FA}\\ \frac{d{\left[\text{ADP}\right]}_{\text{m}}}{dt}=J_{AE}-J_{CAC}-J_{SY}\\ \frac{d{\left[{\text{Ca}}^{2+}\right]}_{\text{m}}}{dt}=\frac{1}{1+\kappa_{Cam}}\times (J_{CU}-J_{HC})\\ \frac{d{\left[\text{Pi}\right]}_{\text{m}}}{dt}=J_{Pi}-J_{SY}-J_{CAC}\\ \frac{d{\left[\text{ADP}\right]}_{\text{c}}}{dt}=J_{Wcrbr}+J_{Wser}+J_{Wb}+J_{GK}+J_{PFK}-J_{AE}-2J_{Ga}\\ \frac{d{\left[\text{Pi}\right]}_{\text{c}}}{dt}=J_{Wcrbr}+J_{Wser}+J_{Wb}-J_{Pi}-J_{Ga}\\ \frac{d{\left[\text{Pyr}\right]}_{\text{m}}}{dt}=J_{Pym}-J_{PDH}\\ \frac{d{\left[\text{Pyr}\right]}_{\text{c}}}{dt}=J_{Ga}+J_{Pyc}-J_{Pym}\\ \frac{d{\left[{\text{NAD}}_{\text{red}}\right]}_{\text{c}}}{dt}=J_{Ga}-J_{MS}-J_{GS}\\ \frac{d[F6P]}{dt}=J_{GK}-J_{PFK}\\ \frac{d[\text{FBP}]}{dt}=J_{PFK}-J_{Al}\\ \frac{d[\text{GAP}]}{dt}=J_{Al}+J_{Ti}-J_{Ga}\\ \frac{d[DHAP]}{dt}=J_{Al}-J_{Ti}\\ \frac{d{\left[{\text{CO}}_{2}\right]}_{\text{m}}}{dt}=J_{PDH}+2J_{CAC}-J_{CO2m}-J_{CAm}\\ \frac{d{\left[{\text{CO}}_{2}\right]}_{\text{c}}}{dt}=J_{Co2m}-J_{CAc}-J_{CO2c}\\ \frac{d{\left[{\text{HCO}}_{3}^{-}\right]}_{\text{m}}}{dt}=J_{CAm}\\ \frac{d{\left[{\text{HCO}}_{3}^{-}\right]}_{\text{c}}}{dt}=J_{CAc}\\ \frac{d[\text{PCr}]}{dt}=-J_{CK}\\ \frac{d[\text{AMP}]}{dt}=-J_{AK} \end{array}$$

The following simulation is used to describe mitochondrial reactions of pyruvate metabolism and coupled [ATP]_c_ production. ATP cycling is allowed by adding an ATP consuming load reaction *J**_W_*.

SIM_OP_^Pyr^:

A16 $$\begin{array}{l} \frac{d(\Delta_{m}\varphi )}{dt}=\frac{1}{{Cm}_{m}\cdot \alpha_{m}}\times \left(\frac{1}{\nu_{s}}J_{SY}+J_{AE}+2J_{CU}+J_{PL}-\nu_{n}J_{NA}-\nu_{f}J_{FA}\right)\\ \frac{d{\left[{\text{H}}^{+}\right]}_{\text{m}}}{dt}=\frac{1}{1+\kappa_{1}^{BU}+\kappa_{2}^{BU}}\times \left(\frac{1}{\nu_{s}}J_{SY}+J_{Pi}+J_{PL}+2J_{HC}+J_{CAm}-\nu_{n}J_{NA}-\nu_{f}J_{FA}\right)\\ \frac{d{\left[{\text{NAD}}_{\text{red}}\right]}_{\text{m}}}{dt}=J_{PDH}+3J_{CAC}+J_{MS}-J_{NA}\\ \frac{d{\left[{\text{FAD}}_{\text{red}}\right]}_{\text{m}}}{dt}=J_{CAC}+J_{GS}-J_{FA}\\ \frac{d{\left[\text{ADP}\right]}_{\text{m}}}{dt}=J_{AE}-J_{CAC}-J_{SY}\\ \frac{d{\left[{\text{Ca}}^{2+}\right]}_{\text{m}}}{dt}=\frac{1}{1+\kappa_{Cam}}\times (J_{CU}-J_{HC})\\ \frac{d{\left[\text{Pi}\right]}_{\text{m}}}{dt}=J_{Pi}-J_{SY}-J_{CAC}\\ \frac{d{\left[\text{ADP}\right]}_{\text{c}}}{dt}=J_{W}-J_{AE}\\ \frac{d{\left[\text{Pi}\right]}_{c}}{dt}=J_{W}-J_{Pi}\\ \frac{d{\left[\text{Pyr}\right]}_{\text{m}}}{dt}=J_{Pym}-J_{PDH}\\ \frac{d{\left[\text{Pyr}\right]}_{\text{c}}}{dt}=J_{Ga}+J_{Pyrc}-J_{Pym}\\ \frac{d{\left[{\text{AcCoA}}_{\text{red}}\right]}_{\text{m}}}{dt}=J_{PDH}-J_{CAC}\\ \frac{d{\left[{\text{CO}}_{2}\right]}_{\text{m}}}{dt}=J_{PDH}+2J_{CAC}-J_{CO2m}-J_{CAm}\\ \frac{d{\left[{\text{CO}}_{2}\right]}_{\text{c}}}{dt}=J_{Co2m}-J_{CAc}-J_{CO2c}\\ \frac{d{\left[{\text{HCO}}_{3}^{-}\right]}_{\text{m}}}{dt}=J_{CAm}\\ \frac{d{\left[{\text{HCO}}_{3}^{-}\right]}_{\text{c}}}{dt}=J_{CAc} \end{array}$$

A17 $$J_{W}=\frac{1}{1+\text{exp}\left(\frac{\left[{\text{Ca}}_{05}^{\text{W}}\right]-{\left[{\text{Ca}}^{2+}\right]}_{\text{c}}}{\left[{\text{S}}_{Ca}^{W}\right]}\right)}L_{W}^{\text{max}}\times (RT\text{ln}(K_{Ld})+A_{ATPc})$$

[Ca_05_^W^]= 0.55 μM, [S*_Ca_**^W^*] = 0.1 μM, *L**_W_*^max^ = 61818 × 10^−8^ (μM/ms) × (mol/J), *K**_Ld_* = 5 × 10^−6^.

The fraction of *J**_W_* associated with OP, is given by (*J**_AE_* − *J**_CAC_*)/*J**_AE_* × *J**_W_*.

### 4. Forced Oscillations

Sinusoidal [Ca^2+^]_c_ oscillations are generated by the function

A18a $$\frac{d{\left[{\text{Ca}}^{2+}\right]}_{c}}{dt}=\frac{A_{0}}{1+e^{\left(\frac{\left[{\text{Ca}}_{\text{amp}}\right]-{\left[{\text{Ca}}^{2+}\right]}_{c0}}{\left[{\text{S}}_{\text{amp}}\right]}\right)}}\text{sin}(\omega t)$$

with

A18b $$\omega ({\left[{\text{Ca}}^{2+}\right]}_{c})=\frac{2\pi }{P_{0}}\gamma \left(1+e^{\left(\frac{{\left[{\text{Ca}}^{2+}\right]}_{c}-[\text{Cp}]}{\left[\text{Sp}\right]}\right)}\right)$$

*A**_0_* = 0.12 μM, [Ca_amp_] = 0.3 μM, [S_amp_] = 0.2 μM, [C_p_] = 0.1 μM, [S_p_] = 0.3 μM, *P**_0_* = 1000 ms, *γ* = 0.433 (dimensionless adjusting factor). In simulations (SIM_GlOx_) constant [Ca^2+^]_c_ of respective equations has to be replaced by the above variable A18a. [Ca^2+^]_c0_ denotes that concentration, which has to be adjusted.

### 5. Maximal Conductances for VMs

*L**_Al_*^max^ = 6,600 × 10^−8^, *L**_Ti_* ^max^ = 105,600 × 10^−8^, *L**_PDH_*^max^ = 16,682 × 10^−8^, *L**_CAC_*^max^ = 3,876 × 10^−8^, *G**_Pym_*^max^ = 110,200, *G**_GS_*^max^ = 881.6, *G**_MS_*^max^ = 11,600, *G**_AE_*^max^ = 185,600, *G**_Pi_*^max^ = 10,579,200, *G**_CU_*^max^ = 5510, *G**_HCE_*^max^ = 7,685, *G**_PL_*^max^ = 2,800. All *L*’s are given in (μM/ms) × (mol/J), all G’s in pS.

### 6. OP as a Two-Flux-System

Conductances *L**_OP_* and *L**_Wop_* and overall *λ* values (*λ*1*_ov_* and (*λ*2*_ov_*, respectively) can be obtained from dissipation functions. OP then can be formulated as a two-flux-system yielding

A19a $$\Phi_{OP}=L_{OP}(\lambda 2_{ov}+1){\left(A_{R}^{o}\right)}^{2}\left(\frac{{\left(-A_{ATPc}\right)}^{2}}{{\left(A_{R}^{o}\right)}^{2}}\frac{\left(\lambda 1_{ov}+1\right)}{\left(\lambda 1_{ov}+1\right)}+2\frac{\left(-A_{ATPc}\right)}{\left(A_{R}^{o}\right)}\frac{1}{\left(\lambda 2_{ov}+1\right)}+1\right)$$

or

A19b $$\Phi_{OP}=L_{OP}(\lambda 2_{ov}+1){\left(A_{R}^{o}\right)}^{2}(x^{2}+2xq_{ov}+1)$$

The normalized dissipation function is given by

A19c $$\Phi_{OP}/(L_{OP}(\lambda 2_{ov}+1)){\left(A_{R}^{o}\right)}^{2}=x^{2}+2xq_{ov}+1$$

*L**_OP_* (*λ*2 *_ov_* + 1) is equivalent to *L**_22_*.

Generally the normalized dissipation function for a two-flux-system is given by

A20a $$\Phi /L_{c}(\lambda 2+1)A2^{2}=x^{2}+2xq+1$$

and the normalized power output by

A20b $$P/L_{c}(\lambda 2+1)A2^{2}=x^{2}+2xq$$

*A**_R_**^o^* represents the input force of OP. It contains both, *A**_NA_* and *A**_FA_*, while the output force is given by −*A**_ATPc_*. To obtain an expression up to the load, −*A**_ATPc_* (= −*A**_W_**^p^*) has to be substituted in Equation A18a by *A**_W_* *^ld^*. All other parameters then have to be defined from a dissipation function, which contains the load reaction.
